# Efficacy and safety of desvenlafaxine in treating patients with major depressive disorder: a network meta-analysis

**DOI:** 10.3389/fnins.2026.1721852

**Published:** 2026-02-06

**Authors:** Lingke Li, Xiaodan Ren, Chenzhao Yuan

**Affiliations:** 1 Luoyang Polytechnic, Luoyang, China; 2 Clinical Center for Immune Disorders, Xinqiao Hospital, Army Medical University, Chongqing, China; 3 Faculty of Nursing, Chiangmai University, Chiangmai, Thailand

**Keywords:** desvenlafaxine, efficacy, majordepressive disorder, network meta-analysis, serotonin-norepinephrine reuptake inhibitor

## Abstract

**Background and objective:**

Desvenlafaxine (DVS), a commonly used serotonin-norepinephrine reuptake inhibitor (SNRI), is widely applied in the treatment of major depressive disorder (MDD). However, the efficacy and safety of different DVS dosages remain controversial. This study aims to systematically evaluate the efficacy and safety of various doses of DVS in treating MDD, providing evidence-based guidance for clinical dose selection.

**Methods:**

A systematic search was conducted in PubMed, Embase, Web of Science, and the Cochrane Library to identify randomized controlled trials (RCTs) comparing different doses of DVS in adult MDD patients. A Bayesian random-effects model was employed for network meta-analysis, and surface under the cumulative ranking curve (SUCRA) was used to assess the overall performance of each dose in terms of efficacy (HAM-D17, CGI-S, MADRS scores) and safety (treatment-emergent adverse events, TEAEs). Publication bias was assessed using funnel plots. All data analyses were performed using R Studio and STATA.

**Results:**

A total of eight RCTs were included. The analysis showed that DVS at 50, 100, 200, and 400 mg/day significantly outperformed placebo in improving HAM-D17, CGI-S, and MADRS scores, with the 200 mg/day dose showing numerically greater improvement than the other doses. Regarding safety, there were no statistically significant differences in adverse event rates between any DVS dose and placebo (*P* > 0.05). According to the SUCRA rankings, DVS 200 mg/day tended to appear higher in the probabilistic ranking, although this reflects relative ordering rather than conclusive evidence of clinical superiority.

**Conclusion:**

DVS at doses ≥ 50 mg/day significantly improves depressive symptoms compared with placebo. Among the evaluated doses, 200 mg/day consistently showed numerically greater improvements while maintaining acceptable tolerability; however, the certainty of dose differences remains limited, and no definitive “optimal” dose can be established based on the current evidence.

## 1 Introduction

Major depressive disorder (MDD) is a highly prevalent and severely disabling psychiatric disorder worldwide. Globally, the incidence of MDD continues to rise, resulting in an increasing economic and societal burden (Cui et al., 2024). Currently, approximately 300 million people worldwide are affected by MDD, making it one of the leading causes of disability (Nagy et al., 2020). Patients with MDD commonly exhibit symptoms such as depressed mood, loss of interest, changes in weight or appetite, and an increased risk of suicide (Rice et al., 2019). However, due to the lack of specific symptoms and objective diagnostic criteria, early identification and prevention of MDD remain major challenges in clinical practice (Kovacs and Lopez-Duran, 2010).

Depression is now understood as a complex neurobiological condition involving dysregulation across multiple interacting neurotransmitter systems. Rather than reflecting simple deficiencies in serotonin or norepinephrine, current evidence suggests that alterations in monoaminergic signaling, glutamatergic modulation, neuroplasticity, and stress-related neurocircuitry all contribute to the pathophysiology of major depressive disorder. This broader conceptualization provides the rationale for evaluating agents such as SNRIs, which modulate both serotonergic and noradrenergic pathways.

Given the pivotal roles of serotonin (5-HT) and norepinephrine (NE) in the pathophysiology of MDD, serotonin-norepinephrine reuptake inhibitors (SNRIs) have been developed to increase the availability of 5-HT and NE in the synaptic cleft. SNRIs have become a key pharmacological option in the treatment of moderate to severe MDD and are often recommended as first-line therapies (Arakawa et al., 2019). Their mechanisms of action mainly include: (1) inhibiting the norepinephrine transporter (NET) to block NE reuptake and increase its concentration in the synaptic cleft (Gould et al., 2007); (2) inhibiting the serotonin transporter (SERT) to increase 5-HT levels (Owens et al., 2008); and (3) enhancing antidepressant efficacy through a dual inhibition mechanism (Sramek et al., 2016). Representative SNRIs include milnacipran, levomilnacipran, duloxetine (DXT), desvenlafaxine (DVS), and venlafaxine.

Despite the availability of various antidepressant treatments, issues such as limited efficacy, poor tolerability, and interindividual variability in response remain common. Therefore, there is an urgent need to further optimize treatment strategies to improve overall efficacy and reduce treatment-related burden. In this context, the present study aims to systematically evaluate the efficacy and safety profiles of different doses of DVS in the treatment of MDD by synthesizing data from existing randomized controlled trials (RCTs) using systematic review and network meta-analysis methods. We aim to clarify the overall benefits and risks associated with each dose level, provide evidence-based recommendations for selecting appropriate DVS dosages in clinical practice, and ultimately promote optimization and personalization of treatment strategies for major depressive disorder. Unlike previous reviews that focused on limited dose comparisons, this study evaluates all fixed-dose regimens simultaneously by adopting a network meta-analytic framework, enabling a more complete assessment of the comparative performance of different DVS dose levels.

## 2 Materials and methods

The protocol was developed prior to data extraction, and the study was subsequently registered in PROSPERO (ID: CRD420251046987). Although the registration occurred after initial screening, all methodological steps followed the predefined protocol, which is now reported transparently for reproducibility.

### 2.1 Retrieval strategy

This study was designed and reported in accordance with the PRISMA-NMA (Preferred Reporting Items for Systematic Reviews and Meta-Analyses incorporating Network Meta-Analyses) guidelines (Hutton et al., 2015). A systematic search was conducted in the PubMed, Embase, Web of Science, and Cochrane Library databases from inception to April 27, 2025. The search terms included “Desvenlafaxine,” “MDD,” and “major depressive disorder,” supplemented by both free-text terms and controlled vocabulary to expand the search. In addition, relevant reviews and conference abstracts were manually screened to identify potentially missed studies. A detailed full-search strategy for each database, including all Boolean operators and MeSH/Emtree terms, has been provided in Supplementary material 1. No language restrictions were applied.

### 2.2 Inclusion and exclusion criteria

#### 2.2.1 Inclusion criteria

(1) Study type: Randomized controlled trial (RCT);

(2) Study population: Adult MDD patients who meet the diagnostic criteria for MDD based on DSM (Hasin et al., 2018) or ICD (Howard et al., 2018);

(3) Intervention: Desvenlafaxine (DVS) at different doses;

(4) Control: Placebo or other doses of DVS;

(5) Outcome measures: At least report changes in HAM-D17, MADRS, or CGI-S scores, as well as the incidence of treatment-emergent adverse events (TEAEs).

#### 2.2.2 Exclusion criteria

(1) Non-randomized studies, observational studies, reviews, case reports;

(2) Incomplete or unextractable data;

(3) Studies with populations affected by comorbid psychiatric disorders that impact the analysis.

### 2.3 Literature selection

Two researchers independently reviewed the articles based on the inclusion and exclusion criteria and cross-validated their findings. In case of any discrepancies, a third researcher was involved to resolve the disagreement and reach a consensus. The retrieved papers were imported into EndNote X9 for management and further examined by the two researchers. Duplicate papers were removed automatically and manually. The remaining articles were independently screened by the researchers according to the predefined inclusion and exclusion criteria, by evaluating the titles, abstracts, and full texts.

### 2.4 Data extraction, quality evaluation and certainty-of-evidence assessment

Data were independently extracted by two researchers, including basic information (authors, publication year, sample size, intervention schemes, etc.), primary outcomes (changes in HAM-D17, CGI-S, MADRS scores), and safety outcomes (incidence of treatment-emergent adverse events, TEAEs). In case of any disagreements, a third researcher was consulted to resolve the issue. The Cochrane Risk of Bias Assessment Tool (RoB 2.0) (Higgins et al., 2011) was used to evaluate the methodological quality of the included RCTs, including random sequence generation, allocation concealment, blinding, completeness of outcome data, and selective reporting. To assess the certainty of the evidence for each major outcome, we conducted a GRADE evaluation (Balshem et al., 2011), considering factors such as risk of bias, inconsistency, imprecision, indirectness, and publication bias.

### 2.5 Statistical analysis

A Bayesian random-effects model was used to conduct a network meta-analysis (NMA), and the Markov Chain Monte Carlo (MCMC) method was employed to estimate the relative effects. The model was run with 4 chains, a burn-in period of 20,000 iterations, and 50,000 iterations in total. Continuous variables (changes in HAM-D17, MADRS, CGI-S) were expressed as mean differences (MD) with 95% confidence intervals (CI), while dichotomous variables (TEAEs) were expressed as relative risks (RR) with 95% CI. The cumulative ranking probability (SUCRA) was used to rank the efficacy and safety of different doses. Consistency of the model was assessed by comparing the DIC values of the consistency and inconsistency models, and the consistency model was chosen when the DIC difference was less than 5. Two separate sensitivity analyses were performed: the first excluded the 25 mg dose, and the second restricted the analysis to 8-week trials. Publication bias was assessed using funnel plots with Egger’s test for asymmetry. All statistical analyses were performed using R Studio (gemtc package) and STATA 17.0 software. The Bayesian model analysis history was provided in Supplementary material 2.

## 3 Results

### 3.1 Results of literature screening

**FIGURE 1:**
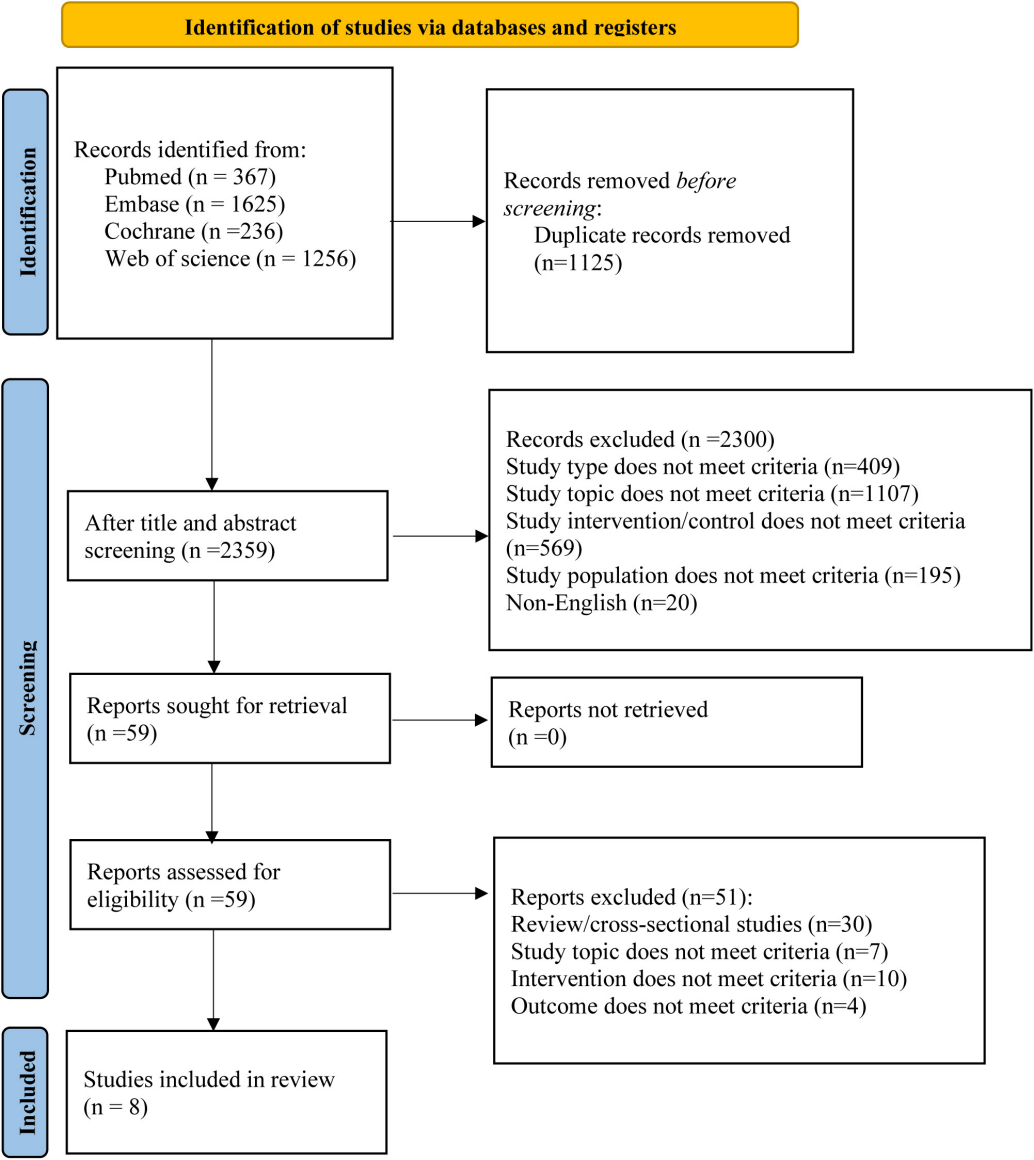
PRISMA flow chart.

A preliminary search identified a total of 3,484 articles, with 2,359 remaining after duplicates were removed. After screening titles and abstracts, 59 articles were included for full-text assessment, and ultimately, 8 RCTs (Boyer et al., 2008; Boyer et al., 2015; Clayton et al., 2013; Clayton et al., 2015; Dunlop et al., 2011; Iwata et al., 2013; Liebowitz et al., 2008; Septien-Velez et al., 2007) met the inclusion criteria. The literature selection process is shown in Figure 1.

### 3.2 Characteristics of included studies

The studies included in this research were all multicenter, randomized controlled trials (RCTs). The DVS doses involved ranged widely, including 25, 50, 100, 200, and 400 mg/day, allowing for a comprehensive reflection of the efficacy and safety of DVS at different dosage levels in the treatment of major depressive disorder (MDD) patients. All studies used standardized depression assessment scales to evaluate efficacy and systematically recorded treatment-emergent adverse events (TEAEs), providing a sufficient and reliable data foundation for this network meta-analysis. Detailed characteristics are shown in Table 1.

**TABLE 1:** Baseline characteristics table.

No.	References	Country	Case source	Disease	Age	Male/ Female	Treatment course	Experimental group intervention (mg/day)	Control group intervention	Outcomes
1	Septien-Velez et al. (2007)	France	Multicenter	MDD	18–75	125/244	8W	Desvenlafaxine 200/400	Placebo	HAM-D17, CGI-S, MADRS, TEAEs
2	Liebowitz et al. (2008)	American	Multicenter	MDD	≥ 18	181/266	8W	Desvenlafaxine 50/100	Placebo	HAM-D17, CGI-S, MADRS, TEAEs
3	Boyer et al. (2008)	France	Multicenter	MDD	≥ 18	147/336	8W	Desvenlafaxine 50/100	Placebo	HAM-D17, CGI-S, MADRS, TEAEs
4	Dunlop et al. (2011)	American	Multicenter	MDD	18–75	146/281	12W	Desvenlafaxine 50	Placebo	HAM-D17, TEAEs
5	Iwata et al. (2013)	Japan	Multicenter	MDD	≥ 18	313/386	8W	Desvenlafaxine 25/50	Placebo	HAM-D17, CGI-S, MADRS, TEAEs
6	Clayton et al. (2013)	American	Multicenter	MDD	40–70	0/432	10W	Desvenlafaxine 50	Placebo	HAM–D17, CGI-S, MADRS, TEAEs
7	Boyer et al. (2015)	France	Multicenter	MDD	18–59	157/391	24W	Desvenlafaxine 50	Placebo	HAM-D17, CGI-S
8	Clayton et al. (2015)	American	Multicenter	MDD	18–56	399/510	8W	Desvenlafaxine 50/100	Placebo	HAM–D17, TEAEs

MDD, Major Depressive Disorder; HAM-D17, 17-item Hamilton Depression Rating Scale; CGI-S, Clinical Global Impressions–Severity of Illness scale; MADRS, Montgomery-Asberg Depression Rating Scale; TEAEs, Treatment-Emergent Adverse Events.

### 3.3 Quality assessment results

**FIGURE 2:**
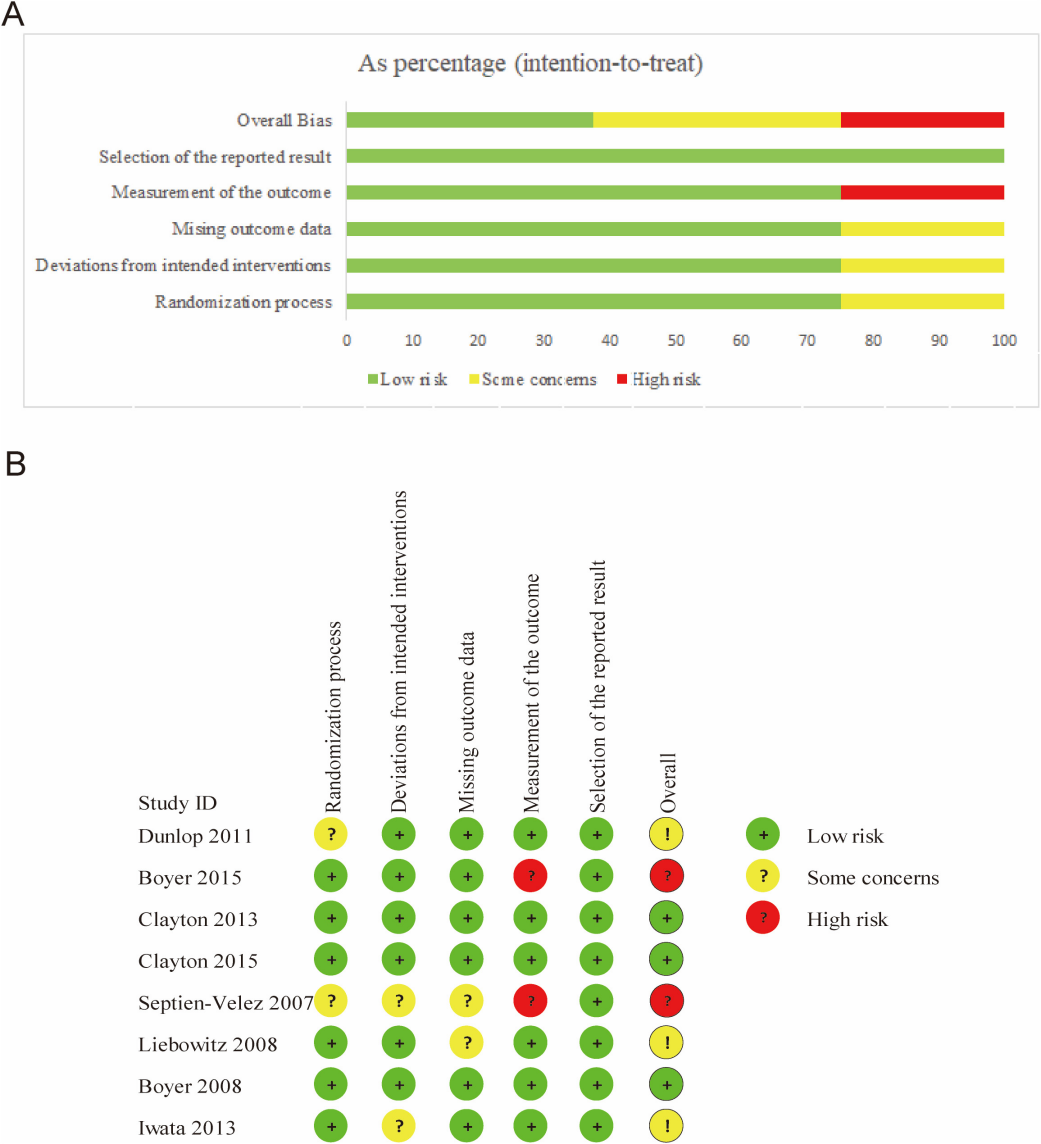
Risk of bias assessment. **(A)** Risk of bias summary. **(B)** Risk of bias graph.

The Cochrane-recommended tool, ROB2, was used to assess the quality of the included studies. Among the 8 trials, 6 clearly described the process of sequence generation, including the use of computer-generated random numbers or random number tables, as well as blinding procedures. However, two studies raised concerns regarding planned intervention differences and missing outcome data. In terms of outcome measurement, two studies were rated as having a high risk of bias. For selective reporting of results, all studies performed well. Regarding overall risk of bias, the distribution was as follows: low risk 37.5%, medium risk 37.5%, and high risk 25%. Based on the quality assessment, the studies varied in quality, and while none were excluded from the analysis, the high risk of bias in some studies must be carefully considered when interpreting the results (Figure 2).

### 3.4 Network meta-analysis

**FIGURE 3:**
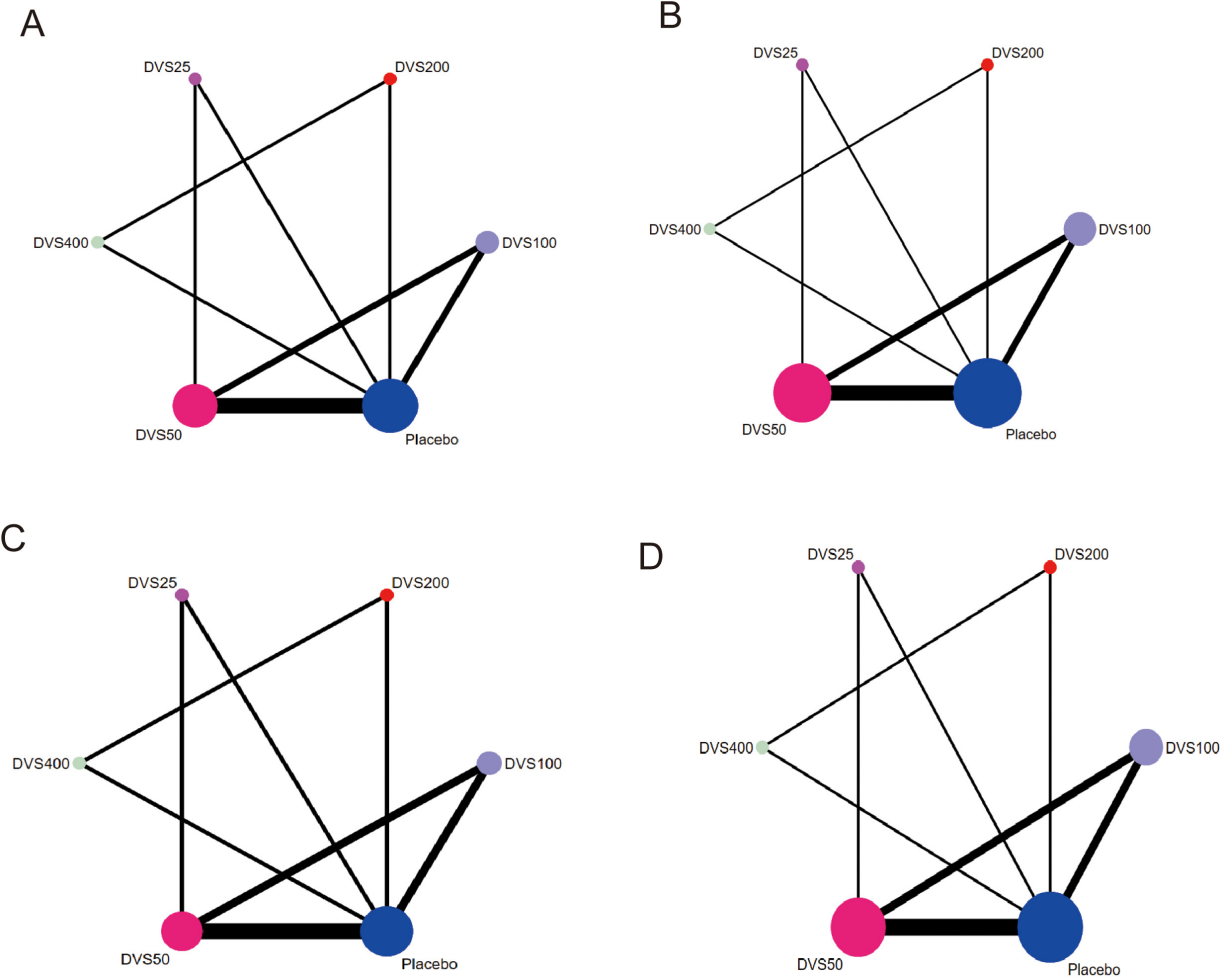
Network diagram. **(A)** CGI-S network diagram; **(B)** HAM-D17 network diagram; **(C)** MADRS network diagram; **(D)** TEAEs network diagram. The network plots display the structure of direct and indirect comparisons. Node size reflects sample size, and edge thickness represents the number of direct comparisons. These diagrams are descriptive in nature and do not imply effect magnitude or certainty.

Figure 3 presents the network diagram of the comparisons between different interventions in this study, providing a visual aid to understand the comparisons between different doses of DVS and placebo. In the diagram, each node represents a specific intervention, such as different doses of Desvenlafaxine (DVS) or placebo. The size of the node is proportional to the number of patients receiving that intervention, with larger nodes indicating a greater number of participants in that intervention group. The lines between nodes represent studies with direct comparisons, and the thickness of the lines reflects the number of studies directly comparing the two interventions. The thicker the line, the more data are available for that comparison, and the stronger the evidence for that direct comparison. The network diagram allows for an intuitive understanding of the richness of direct or indirect comparisons between doses, helping to assess the connectivity of the network meta-analysis and the robustness of the overall evidence base.

### 3.5 Efficacy analysis

#### 3.5.1 HAM-D17

A total of 8 studies (Boyer et al., 2008; Boyer et al., 2015; Clayton et al., 2013; Clayton et al., 2015; Dunlop et al., 2011; Iwata et al., 2013; Liebowitz et al., 2008; Septien-Velez et al., 2007) reported changes in the HAM-D17 scores. The analysis showed that compared with placebo, the effect size for 25 mg/day was MD = -0.71, 95% CI = (−1.79 to 0.38), which did not show statistical significance. However, 50 mg/day [MD = −1.99, 95% CI = (−2.48 to −1.51)], 100 mg/day [MD = −2.14, 95% CI = (−2.91 to −1.36)], 200 mg/day [MD = −3.29, 95% CI = (−5.36 to −1.24)], and 400 mg/day [MD = −2.80, 95% CI = (−4.84 to −0.74)] all significantly reduced the HAM-D17 scores. In comparisons between different doses, only 50 mg/day, 100 mg/day and 200 mg/day showed statistically significant differences when compared to 25 mg/day, with no significant differences observed between other doses (see Table 2 for details).

**TABLE 2:** HAM-D17 league table (MD 95%CI).

DVS25	DSV50	DVS100	DVS200	DVS400	Placebo
**1.28 (0.2, 2.37)**	–	–	–	–	–
**1.43 (0.14, 2.72)**	0.15 (−0.63, 0.92)				
**2.59 (0.26, 4.92)**	1.3 (−0.81, 3.42)	1.15 (−1.05, 3.37)			
2.09 (−0.23, 4.4)	0.8 (−1.3, 2.91)	0.66 (−1.53, 2.85)	−0.5 (−2.56, 1.56)		
−0.71 (−1.79, 0.38)	−**1.99 (**−**2.48,** −**1.51)**	−**2.14 (**−**2.91,** −**1.36)**	−**3.29 (**−**5.36,** −**1.24)**	−**2.8 (**−**4.84,** −**0.74)**	

Bold values indicate statistically significant.

#### 3.5.2 CGI-S

A total of 6 studies (Boyer et al., 2008; Boyer et al., 2015; Clayton et al., 2013; Iwata et al., 2013; Liebowitz et al., 2008; Septien-Velez et al., 2007) reported changes in the CGI-S scores. The analysis showed that compared with placebo, DVS doses of 25 mg/day [MD = −0.27, 95% CI = (−0.45 to −0.1)], 50 mg/day [MD = −0.37, 95% CI = (−0.47 to −0.28)], 100 mg/day [MD = −0.38, 95% CI = (−0.59 to −0.17)], 200 mg/day (MD = −0.6, 95% CI = [−0.91 to −0.29)], and 400 mg/day [MD = −0.4, 95% CI = (−0.69 to −0.11)] all significantly reduced the CGI-S scores, and all reached statistical significance. No significant differences were observed in the comparisons between different doses (see Table 3 for details).

**TABLE 3:** CGI-S league table (MD 95%CI).

DVS25	DVS50	DVS100	DVS200	DVS400	Placebo
0.1 (−0.07, 0.28)	–	–	–	–	–
0.11 (−0.16, 0.38)	0.01 (−0.21, 0.22)				
0.33 (−0.03, 0.69)	0.23 (−0.1, 0.55)	0.22 (−0.16, 0.6)			
0.13 (−0.21, 0.46)	0.03 (−0.28, 0.33)	0.02 (−0.33, 0.37)	−0.2 (−0.54, 0.13)		
−**0.27 (**−**0.45,** −**0.1)**	−**0.37 (**−**0.47,** −**0.28)**	−**0.38 (**−**0.59,** −**0.17)**	−**0.6 (**−**0.91,** −**0.29)**	−**0.4 (**−**0.69,** −**0.11)**	

Bold values indicate statistically significant.

#### 3.5.3 MADRS

A total of 5 studies (Boyer et al., 2008; Clayton et al., 2013; Iwata et al., 2013; Liebowitz et al., 2008; Septien-Velez et al., 2007) reported changes in the MADRS scores. The analysis showed that compared with placebo, DVS 25 mg/day [MD = −1.27, 95% CI = (−2.79 to 0.27)] did not show a statistically significant difference, while DVS 50 mg/day [MD = −2.59, 95% CI = (−3.56 to −1.64)], DVS 100 mg/day [MD = −2.85, 95% CI = (−4.43 to −1.29)], DVS 200 mg/day [MD = −4.29, 95% CI = (−6.52 to −2.05)], and DVS 400 mg/day [MD = −3.60, 95% CI = (−5.80 to −1.40)] all significantly reduced the MADRS scores. In the comparisons between different doses, only 25 mg/day compared to 200 mg/day showed a statistically significant difference (see Table 4 for details).

**TABLE 4:** MADRS league table (MD 95%CI).

DVS25	DVS50	DVS100	DVS200	DVS400	Placebo
1.33 (−0.19, 2.85)	–	–	–	–	–
1.59 (−0.5, 3.68)	0.26 (−1.32, 1.85)				
**3.02 (0.3, 5.73)**	1.7 (−0.75, 4.14)	1.44 (−1.3, 4.15)			
2.34 (−0.34, 5.02)	1.01 (−1.39, 3.4)	0.75 (−1.95, 3.45)	−0.69 (−3.22, 1.85)		
−1.27 (−2.79, 0.27)	−**2.59 (**−**3.56,** −**1.64)**	−**2.85 (**−**4.43,** −**1.29)**	−**4.29 (**−**6.52,** −**2.05)**	−**3.6 (**−**5.8,** −**1.4)**	

Bold values indicate statistically significant.

### 3.6 Safety analysis

A total of 7 studies (Boyer et al., 2008; Clayton et al., 2013; Clayton et al., 2015; Dunlop et al., 2011; Iwata et al., 2013; Liebowitz et al., 2008; Septien-Velez et al., 2007) reported the incidence of treatment-emergent adverse events (TEAEs). The analysis showed that compared with placebo, DVS 25 mg/day [RR = 1.11, 95% CI = (0.75–1.63)], DVS 200 mg/day [RR = 1.20, 95% CI = (0.78–1.85)], and DVS 400 mg/day [RR = 1.28, 95% CI = (0.84–1.97)] did not show statistically significant differences. However, DVS 50 mg/day [RR = 1.25, 95% CI = (1.04–1.49)] and DVS 100 mg/day [RR = 1.30, 95% CI = (1.02–1.66)] showed statistically significant differences compared to placebo. No significant differences were observed in comparisons between different doses (see Table 5 for details).

**TABLE 5:** TEAEs league table (RR 95%CI).

DVS25	DVS50	DVS100	DVS200	DVS400	Placebo
0.89 (0.61, 1.31)	–	–	–	–	–
0.85 (0.55, 1.32)	0.96 (0.76, 1.21)				
0.92 (0.52, 1.65)	1.04 (0.65, 1.66)	1.08 (0.66, 1.78)			
0.86 (0.48, 1.54)	0.97 (0.61, 1.55)	1.01 (0.62, 1.66)	0.94 (0.61, 1.43)		
1.11 (0.75, 1.63)	**1.25 (1.04, 1.49)**	**1.30 (1.02, 1.66)**	1.20 (0.78, 1.85)	1.28 (0.84, 1.97)	

Bold values indicate statistically significant.

### 3.7 Ranking results

**FIGURE 4:**
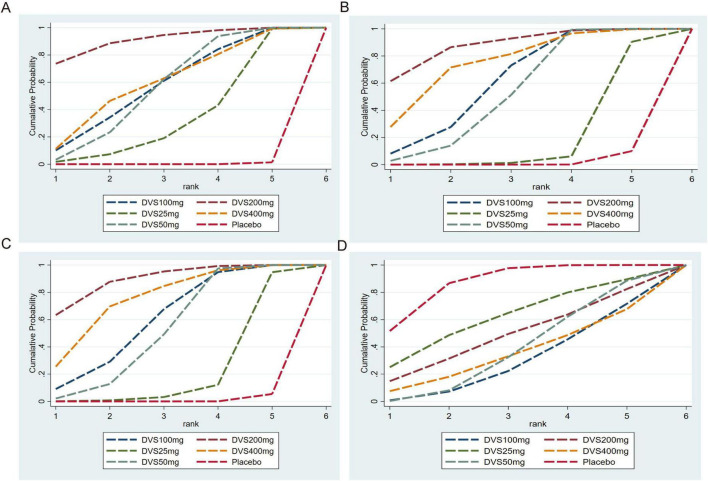
SUCRA-based treatment effectiveness ranking chart. **(A)** CGI-S merges SUCRA; **(B)** HAM-D17 merges SUCRA; **(C)** MADRS merges SUCRA; **(D)** TEAEs merges SUCRA. SUCRA values represent the probability distribution of ranks within the network and indicate relative ordering rather than clinical superiority. These rankings should be interpreted alongside effect estimates and credible intervals.

The cumulative efficacy ranking showed that DVS 200, 400, and 100 mg/day ranked in the top three for reducing HAM-D17, CGI-S, and MADRS scores. The top three doses with the lowest incidence of treatment-emergent adverse events (TEAEs) were placebo, DVS 25 mg/day, and DVS 200 mg/day (see Figure 4 for details). As SUCRA values summarize probabilistic ranking rather than clinical superiority, all SUCRA results were interpreted cautiously and in conjunction with the corresponding effect estimates.

### 3.8 Sensitivity analysis

To assess the robustness of the findings, two sensitivity analyses were conducted. The first analysis excluded the 25 mg dose from the network meta-analysis. For the TEAE outcome, this exclusion removed the statistically significant difference previously observed between the 50 mg, 100 mg doses, and placebo. The ranking of treatments also remained mostly unchanged, with the top three treatments still consistent. The second sensitivity analysis restricted the analysis to 8-week trials. Similar to the first analysis, the results for TEAE outcomes showed no statistically significant difference, and the treatment rankings remained stable with only slight changes in the lower ranks. For all other outcomes, both sensitivity analyses did not result in any meaningful changes, either in statistical significance or in the treatment rankings (see Supplementary material 3).

### 3.9 Publication bias and inconsistency testing

To assess publication bias, Egger’s test was performed for all outcomes. The results showed no significant asymmetry, with the following *p*-values: HAM-D17 (*p* = 0.35), CGI-S (*p* = 0.90), MADRS (*p* = 0.75), and TEAEs (*p* = 0.75). Therefore, no evidence of significant publication bias was found. Funnel plots and Egger’s test results are presented descriptively in Supplementary material 2. Despite the presence of closed loops in the data, node-splitting analysis could not be performed because there are no comparisons to assess for inconsistency, and therefore no consistency testing results were provided.

### 3.10 GRADE certainty-of-evidence assessment

For each major outcome in the network meta-analysis, a GRADE assessment was conducted to evaluate the certainty of the evidence. The evidence for HAM-D17 was rated as moderate due to a serious risk of bias, with no issues in inconsistency, indirectness, or imprecision, and no publication bias detected. The evidence for CGI-S was rated as low due to both serious inconsistency and serious risk of bias, while no concerns regarding indirectness, imprecision, or publication bias were found. For MADRS and TEAEs, the evidence was rated as high, with no issues in bias, inconsistency, indirectness, imprecision, or publication bias. The full GRADE assessment for each outcome is summarized in Table 6.

**TABLE 6:** GRADE certainty-of-evidence assessment.

Outcome	Risk of bias	Inconsistency	Indirectness	Imprecision	Publication bias	GRADE rating
HAM-D17	Serious	Not serious	Not serious	Not serious	None	⨁⨁⨁◯ Moderate
CGI-S	Serious	Serious	Not serious	Not serious	None	⨁⨁◯◯ Low
MADRS	Not serious	Not serious	Not serious	Not serious	None	⨁⨁⨁⨁ High
TEAEs	Not serious	Not serious	Not serious	Not serious	None	⨁⨁⨁⨁ High

## 4 Discussion

This study is the first to systematically assess the efficacy and safety gradient of different fixed doses of Desvenlafaxine (DVS) for treating adult major depressive disorder (MDD) based on a network meta-analysis framework. The results indicate that DVS at doses ≥ 50 mg/day exhibits significant antidepressant effects, with the 200 mg/day dose showing a tendency toward greater improvement in HAM-D17, CGI-S, and MADRS scores, rather than definitive evidence of the best improvement, and no statistical difference in adverse event incidence compared to the placebo group (*P* > 0.05). This finding aligns with the dual-action mechanism of SNRIs—through dose-dependent inhibition of 5-HT and NE reuptake (NET inhibition rate of approximately 80%, SERT inhibition rate of approximately 30%), effectively increasing neurotransmitter concentrations in the synaptic gap (Gould et al., 2007; Sramek et al., 2016). Notably, although 200 mg/day appeared higher in the SUCRA probability rankings, SUCRA reflects relative ordering rather than definitive evidence of an optimal NET/SERT balance, and therefore should be interpreted cautiously. This provides incremental value by summarizing relative dose ordering rather than resolving definitive dose-related differences.

The study’s results make an important revision to the traditional dose-response understanding. While early RCT studies (Thase et al., 2009) suggested no additional benefit for DVS doses > 50 mg/day, this study, by integrating evidence from 8 RCTs with 4,314 patients, indicates that 200 mg/day shows a numerically larger standardized mean difference compared to 50 mg/day, although credible intervals overlap and the certainty of this difference is limited. This difference may stem from methodological advances: the network meta-analysis overcomes the limitations of traditional pairwise comparisons and quantifies the dose-response gradient using Bayesian models, while previous studies may have produced Type II errors due to sample size limitations. Additionally, this study found that the risk of adverse events significantly increased in the 50–100 mg/day dose group (RR = 1.25–1.30), but no dose-dependent risk increase was observed in the 200–400 mg/day groups, which is closely related to DVS’s unique pharmacokinetic (Naseeruddin et al., 2023) characteristics. Its low protein binding rate ( < 30%) and non-CYP450-dependent metabolic pathway (Naseeruddin et al., 2023) may reduce fluctuations in blood drug concentration at higher doses, maintaining a good safety threshold.

Sensitivity analyses further validated the robustness of our findings. First, when the 25 mg/d dose was excluded, the difference in adverse event risk between the 50 mg/d and 100 mg/d treatment groups and the placebo group disappeared, suggesting that the effect of the lower dose may have substantially influenced the results. Second, restricting the analysis to the 8-week treatment period did not substantially alter the findings, suggesting that efficacy and safety differences between dose groups remained consistent despite variations in treatment duration. These sensitivity analysis results strengthen our confidence in the primary conclusions.

The GRADE assessment results further deepen our understanding of these conclusions. For the HAM-D17 analysis, although our study identified significant differences in therapeutic efficacy, the GRADE assessment indicates that the evidence quality for this conclusion is moderate. This suggests we should exercise caution regarding the stability of this conclusion. The reason lies in the risk of bias in randomization and blinding observed in some studies, which reduces the reliability of the results. For CGI-S outcomes, evidence quality was rated as low, primarily due to substantial heterogeneity and risk of bias across studies. This underscores the need for further high-quality data to confirm these findings. In contrast, evidence quality for MADRS and TEAEs was rated as high, indicating relatively reliable conclusions that support the consistency of DVS in both efficacy and safety.

In clinical practice, this study provides key evidence for dose selection. While the EMA guidelines recommend 50 mg/day as the starting dose (Oliva et al., 2021), our SUCRA ranking suggests that 200 mg/day tends to rank relatively higher, but SUCRA does not establish clinical superiority or a definitive efficacy–safety balance. Dose escalation to 200 mg/day may be considered for patients who do not respond adequately to 50 mg/day, but this must be individualized, as ranking probabilities alone cannot justify dosing recommendations. Notably, doses > 200 mg/day yielded only small numerical improvements, and the clinical relevance of this difference remains uncertain, suggesting that clinical decisions should weigh the small incremental effects against potential cost increases.

The limitations of this study suggest future research directions: First, the median treatment duration of the included RCTs was 6 weeks (IQR: 4–8 weeks), which limits the ability to assess the long-term effects of treatment on relapse prevention and functional recovery. A major limitation is that the included trials differed in population characteristics and treatment durations, yet did not provide sufficiently consistent stratified data to support meaningful subgroup or sensitivity analyses. As a result, the network estimates should be interpreted with caution, and future trials with harmonized reporting are needed to clarify potential effect modifiers. Second, there were differences in the adverse event recording standards across studies (e.g., TEAEs definitions ranged from ≥ 5 to ≥ 10% incidence), which may affect the precision of safety assessments. Additionally, the lack of pharmacogenomic subgroup analyses (such as CYP2D6 rapid/slow metabolizer status) limits the development of personalized dosing strategies (Bousman et al., 2023). Future studies should use a unified core outcome set (COS) and employ individual patient data meta-analysis (IPDMA) to explore the sources of heterogeneity in dose-response relationships.

It is important to emphasize that SUCRA is a descriptive probabilistic tool and does not quantify effect magnitude, clinical superiority, or certainty of evidence. Therefore, all SUCRA findings in this study are interpreted cautiously and always in conjunction with effect sizes, credible intervals, heterogeneity, inconsistency, and risk-of-bias assessments.

## 5 Conclusion

Based on the currently available RCT evidence synthesized in this network meta-analysis, DVS doses ≥ 50 mg/day are effective for the treatment of adult MDD compared with placebo. Among the evaluated regimens, 200 mg/day often showed numerically greater improvements in depressive symptoms while maintaining acceptable tolerability; however, the certainty of dose differences is limited, and no definitive optimal dose can be established. These findings provide comparative information that may inform individualized dosing decisions, but further high-quality, long-term, and head-to-head trials are needed before any changes to clinical guideline recommendations can be confidently proposed.

## Data Availability

The original contributions presented in this study are included in this article/Supplementary material, further inquiries can be directed to the corresponding author.
