# Supplementary material for: Efficacy and safety of desvenlafaxine in treating patients with major depressive disorder: a network meta-analysis
Source: Front Neurosci. 2026 Feb 6;20:1721852. doi: 10.3389/fnins.2026.1721852 (PMC12920435; doi:10.3389/fnins.2026.1721852)
Supplement: Supplementary file 1 [file Data_Sheet_1.docx]

**PUBMED检索历史**

| Search number | Query | | Results |
| --- | --- | --- | --- |
| 4 | ((((((((((((((((((((((((((((((((Depression[Title/Abstract]) OR (Depressive Disorder[Title/Abstract])) OR (Depressive Disorders[Title/Abstract])) OR (Depressive Symptoms[Title/Abstract])) OR (Depressive Symptom[Title/Abstract])) OR (Depressive Syndromes[Title/Abstract])) OR (Syndrome, Depressive[Title/Abstract])) OR (Syndromes, Depressive[Title/Abstract])) OR (Symptom, Depressive[Title/Abstract])) OR (depressive syndrome[Title/Abstract])) OR (depressive disease[Title/Abstract])) OR (depressive episode[Title/Abstract])) OR (depressive illness[Title/Abstract])) OR (depressive personality disorder[Title/Abstract])) OR (depressive state[Title/Abstract])) OR (depressivity[Title/Abstract])) OR (Disorder, Depressive[Title/Abstract])) OR (Disorders, Depressive[Title/Abstract])) OR (Neurosis, Depressive[Title/Abstract])) OR (Depressive Neuroses[Title/Abstract])) OR (Depressive Neurosis[Title/Abstract])) OR (Depressions, Neurotic[Title/Abstract])) OR (Neurotic Depressions[Title/Abstract])) OR (Neuroses, Depressive[Title/Abstract])) OR (Depressions, Endogenous[Title/Abstract])) OR (Endogenous Depressions[Title/Abstract])) OR (Melancholia[Title/Abstract])) OR (Melancholias[Title/Abstract])) OR (Depressions, Unipolar[Title/Abstract])) OR (Unipolar Depressions[Title/Abstract])) OR ("Depressive Disorder"[Mesh])) OR ("Depression"[Mesh])) AND (("Desvenlafaxine Succinate"[Mesh]) OR ((((((((((((((((((Desvenlafaxine Succinate[Title/Abstract]) OR (Desvenlafaxine[Title/Abstract])) OR (Succinate, Desvenlafaxine[Title/Abstract])) OR (Succinate, O-desmethylvenlafaxine[Title/Abstract])) OR (desmethylvenlafaxine Succinate Monohydrate[Title/Abstract])) OR (Monohydrate, O-desmethylvenlafaxine Succinate[Title/Abstract])) OR (desmethylvenlafaxine Succinate Monohydrate[Title/Abstract])) OR (Succinate Monohydrate, O-desmethylvenlafaxine[Title/Abstract])) OR (2-(1-hydroxycyclohexyl)-2-((4-hydroxyphenyl)ethyl)dimethylammonium 3-carboxypropanoate monohydrate[Title/Abstract])) OR (4-(2-(dimethylamino)-1-(1-hydroxycyclohexyl)ethyl)phenol[Title/Abstract])) OR (desmethylvenlafaxine[Title/Abstract])) OR (Pristiq[Title/Abstract])) OR (4 (2 (dimethylamino) 1 (1 hydroxycyclohexyl) ethyl) phenol[Title/Abstract])) OR (4 (2 dimethylamino 1 (1 hydroxycyclohexyl) ethyl) phenol[Title/Abstract])) OR (ellefore[Title/Abstract])) OR (khedezla[Title/Abstract])) OR (o norvenlafaxine[Title/Abstract])) OR (pristiqs[Title/Abstract]))) | | 367 |
| 3 | "Depressive Disorder"[Mesh] | Most Recent | 135,441 |
| 2 | "Depression"[Mesh] | Most Recent | 163,554 |
| 1 | "Desvenlafaxine Succinate"[Mesh] | Most Recent | 385 |

**EMBASE检索历史**

| No. | Query | Results |
| --- | --- | --- |
| #3 | #1 AND #2 | 1625 |
| #2 | depression:ab,ti OR 'depressive disorder':ab,ti OR 'depressive disorders':ab,ti OR 'depressive symptoms':ab,ti OR 'depressive symptom':ab,ti OR 'depressive syndromes':ab,ti OR 'syndrome, depressive':ab,ti OR 'syndromes, depressive':ab,ti OR 'symptom, depressive':ab,ti OR 'depressive syndrome':ab,ti OR 'depressive disease':ab,ti OR 'depressive episode':ab,ti OR 'depressive illness':ab,ti OR 'depressive personality disorder':ab,ti OR 'depressive state':ab,ti OR depressivity:ab,ti OR 'disorder, depressive':ab,ti OR 'disorders, depressive':ab,ti OR 'neurosis, depressive':ab,ti OR 'depressive neuroses':ab,ti OR 'depressive neurosis':ab,ti OR 'depressions, neurotic':ab,ti OR 'neurotic depressions':ab,ti OR 'neuroses, depressive':ab,ti OR 'depressions, endogenous':ab,ti OR 'endogenous depressions':ab,ti OR melancholia:ab,ti OR melancholias:ab,ti OR 'depressions, unipolar':ab,ti OR 'unipolar depressions':ab,ti OR 'depression'/exp | 986552 |
| #1 | desvenlafaxine:ab,ti OR 'succinate, desvenlafaxine':ab,ti OR 'succinate, o-desmethylvenlafaxine':ab,ti OR 'monohydrate, o-desmethylvenlafaxine succinate':ab,ti OR 'desmethylvenlafaxine succinate monohydrate':ab,ti OR 'succinate monohydrate, o-desmethylvenlafaxine':ab,ti OR pristiq:ab,ti OR ellefore:ab,ti OR khedezla:ab,ti OR 'o norvenlafaxine':ab,ti OR pristiqs:ab,ti OR 'desvenlafaxine'/exp | 3360 |

**WEB OF SCIENCE检索历史**

| # | 检索式 | 检索结果 |
| --- | --- | --- |
| 1 | Desvenlafaxine Succinate (主题) OR Desvenlafaxine (主题) OR Succinate, Desvenlafaxine (主题) OR Succinate, O-desmethylvenlafaxine (主题) OR Monohydrate, O-desmethylvenlafaxine Succinate (主题) OR Succinate Monohydrate, O-desmethylvenlafaxine (主题) OR desmethylvenlafaxine (主题) OR Pristiq (主题) OR ellefore (主题) OR khedezla (主题) OR o norvenlafaxine (主题) OR pristiqs (主题) | 1523 |
| 2 | Depression (主题) OR Depressive Disorder (主题) OR Depressive Disorders (主题) OR Depressive Symptoms (主题) OR Depressive Symptom (主题) OR Depressive Syndromes (主题) OR desmethylvenlafaxine (主题) OR Syndrome, Depressive (主题) OR Syndromes, Depressive (主题) OR Symptom, Depressive (主题) OR depressive syndrome (主题) OR depressive disease (主题) OR depressive episode (主题) OR depressive illness (主题) OR depressive personality disorder (主题) OR depressive state (主题) OR depressivity (主题) OR Disorder, Depressive (主题) OR Disorders, Depressive (主题) OR Neurosis, Depressive (主题) OR Depressive Neuroses (主题) OR Depressive Neurosis (主题) OR Depressions, Neurotic (主题) OR Neurotic Depressions (主题) OR Neuroses, Depressive (主题) OR Depressions, Endogenous (主题) OR Endogenous Depressions (主题) OR Melancholia (主题) OR Melancholias (主题) OR Depressions, Unipolar (主题) OR Unipolar Depressions (主题) | 1356897 |
| 3 | #1 AND #2 | 1256 |

**COCHRANE检索历史 236**

**ID Search**

**#1 MeSH descriptor: [Desvenlafaxine Succinate] explode all trees**

**#2 (Desvenlafaxine Succinate):ti,ab,kw OR (Desvenlafaxine):ti,ab,kw OR (Succinate, Desvenlafaxine):ti,ab,kw OR (Succinate, O-desmethylvenlafaxine):ti,ab,kw OR (Monohydrate, O-desmethylvenlafaxine Succinate):ti,ab,kw**

**#3 (Succinate Monohydrate, O-desmethylvenlafaxine):ti,ab,kw OR (desmethylvenlafaxine):ti,ab,kw OR (Pristiq):ti,ab,kw**

**#4 (ellefore):ti,ab,kw OR (khedezla):ti,ab,kw OR (o norvenlafaxine):ti,ab,kw**

**#5 (pristiqs):ti,ab,kw**

**#6 #1 OR #2 OR #3 OR #4 OR #5**

**#7 MeSH descriptor: [Depression] explode all trees**

**#8 MeSH descriptor: [Depressive Disorder] explode all trees**

**#9 (Depression):ti,ab,kw OR (Depressive Disorder):ti,ab,kw OR (Depressive Disorders):ti,ab,kw OR (Depressive Symptoms):ti,ab,kw OR (Depressive Symptom):ti,ab,kw**

**#10 (Depressive Syndromes):ti,ab,kw OR (Syndrome, Depressive):ti,ab,kw OR (Syndromes, Depressive):ti,ab,kw OR (Symptom, Depressive):ti,ab,kw OR (depressive syndrome):ti,ab,kw**

**#11 (depressive disease):ti,ab,kw OR (depressive episode):ti,ab,kw OR (depressive illness):ti,ab,kw OR (depressive personality disorder):ti,ab,kw OR (depressive state):ti,ab,kw**

**#12 (depressivity):ti,ab,kw OR (Disorder, Depressive):ti,ab,kw OR (Disorders, Depressive):ti,ab,kw OR (Neurosis, Depressive):ti,ab,kw OR (Depressive Neuroses):ti,ab,kw**

**#13 (Depressive Neurosis):ti,ab,kw OR (Depressions, Neurotic):ti,ab,kw OR (Neurotic Depressions):ti,ab,kw OR (Neuroses, Depressive):ti,ab,kw OR (Depressions, Endogenous):ti,ab,kw**

**#14 (Endogenous Depressions):ti,ab,kw OR (Melancholia):ti,ab,kw OR (Melancholias):ti,ab,kw OR (Depressions, Unipolar):ti,ab,kw OR (Unipolar Depressions):ti,ab,kw**

**#15 #7 OR #8 OR #9 OR #10 OR #11 OR #12 OR #13 OR #14**

**#16 #6 AND #15**
