# Supplementary material for: Efficacy and safety of desvenlafaxine in treating patients with major depressive disorder: a network meta-analysis
Source: Front Neurosci. 2026 Feb 6;20:1721852. doi: 10.3389/fnins.2026.1721852 (PMC12920435; doi:10.3389/fnins.2026.1721852)

# HAM-D17

## Inconsistency Modeling

> modelume<-mtc.model(network,type="ume",n.chain=4,likelihood="normal",link="identity",linearModel="random")

> resultsume <- mtc.run(modelume, n.adapt = 20000, n.iter = 50000, thin = 1)

Compiling model graph

Resolving undeclared variables

Allocating nodes

Graph information:

Observed stochastic nodes: 21

Unobserved stochastic nodes: 29

Total graph size: 290

Initializing model

|++++++++++++++++++++++++++++++++++++++++++++++++++| 100%

|**************************************************| 100%

> summary(resultsume)

Results on the Mean Difference scale

Iterations = 20001:70000

Thinning interval = 1

Number of chains = 4

Sample size per chain = 50000

1. Empirical mean and standard deviation for each variable,

plus standard error of the mean:

Mean SD Naive SE Time-series SE

d.Desvenlafaxine100.Desvenlafaxine50 0.1398 0.5468 0.0012228 0.006086

d.Desvenlafaxine100.Placebo 2.1795 0.5506 0.0012312 0.006446

d.Desvenlafaxine200.Desvenlafaxine400 0.5223 1.1872 0.0026547 0.017056

d.Desvenlafaxine200.Placebo 3.3230 1.1931 0.0026679 0.017972

d.Desvenlafaxine25.Desvenlafaxine50 -1.0442 0.8472 0.0018944 0.008618

d.Desvenlafaxine25.Placebo 0.4542 0.8499 0.0019003 0.008769

d.Desvenlafaxine50.Placebo 2.1158 0.4807 0.0010748 0.004040

sd.d 0.4191 0.3817 0.0008535 0.006481

2. Quantiles for each variable:

2.5% 25% 50% 75% 97.5%

d.Desvenlafaxine100.Desvenlafaxine50 -0.94053 -0.20527 0.1404 0.4852 1.206

d.Desvenlafaxine100.Placebo 1.11873 1.83253 2.1716 2.5191 3.276

d.Desvenlafaxine200.Desvenlafaxine400 -1.84592 -0.24308 0.5398 1.2800 2.860

d.Desvenlafaxine200.Placebo 1.02462 2.54724 3.3243 4.0964 5.684

d.Desvenlafaxine25.Desvenlafaxine50 -2.70393 -1.56148 -1.0414 -0.5308 0.593

d.Desvenlafaxine25.Placebo -1.19659 -0.06852 0.4449 0.9787 2.104

d.Desvenlafaxine50.Placebo 1.15966 1.83127 2.1174 2.4058 3.046

sd.d 0.01258 0.14315 0.3213 0.5799 1.411

-- Model fit (residual deviance):

Dbar pD DIC

18.90395 16.64723 35.55119

21 data points, ratio 0.9002, I^2 = 0%

## Consistency Modeling

> model<-mtc.model(network,type="consistency",n.chain=4,likelihood="normal",link="identity",linearModel="random")

> results <- mtc.run(model, n.adapt = 20000, n.iter = 50000, thin = 1)

Compiling model graph

Resolving undeclared variables

Allocating nodes

Graph information:

Observed stochastic nodes: 21

Unobserved stochastic nodes: 27

Total graph size: 324

Initializing model

|++++++++++++++++++++++++++++++++++++++++++++++++++| 100%

|**************************************************| 100%

> summary(results)

Results on the Mean Difference scale

Iterations = 20001:70000

Thinning interval = 1

Number of chains = 4

Sample size per chain = 50000

1. Empirical mean and standard deviation for each variable,

plus standard error of the mean:

Mean SD Naive SE Time-series SE

d.Placebo.Desvenlafaxine100 -2.1554 0.4577 0.0010234 0.006085

d.Placebo.Desvenlafaxine200 -3.3110 1.1246 0.0025147 0.018491

d.Placebo.Desvenlafaxine25 -0.7249 0.6660 0.0014891 0.007418

d.Placebo.Desvenlafaxine400 -2.7943 1.1388 0.0025463 0.017708

d.Placebo.Desvenlafaxine50 -2.0042 0.2948 0.0006591 0.002999

sd.d 0.3247 0.2727 0.0006097 0.004566

2. Quantiles for each variable:

2.5% 25% 50% 75% 97.5%

d.Placebo.Desvenlafaxine100 -3.05708 -2.4476 -2.1507 -1.8587 -1.2615

d.Placebo.Desvenlafaxine200 -5.49634 -4.0673 -3.3147 -2.5656 -1.0761

d.Placebo.Desvenlafaxine25 -2.05056 -1.1411 -0.7235 -0.3044 0.5792

d.Placebo.Desvenlafaxine400 -5.00681 -3.5409 -2.8006 -2.0441 -0.5119

d.Placebo.Desvenlafaxine50 -2.58468 -2.1910 -2.0042 -1.8175 -1.4249

sd.d 0.01079 0.1225 0.2606 0.4528 1.0215

-- Model fit (residual deviance):

Dbar pD DIC

17.66255 14.60299 32.26554

21 data points, ratio 0.8411, I^2 = 0%

## Heterogeneity Analysis

> result.anohe <- mtc.anohe(network)

Compiling model graph

Resolving undeclared variables

Allocating nodes

Graph information:

Observed stochastic nodes: 21

Unobserved stochastic nodes: 21

Total graph size: 213

Initializing model

|**************************************************| 100%

Compiling model graph

Resolving undeclared variables

Allocating nodes

Graph information:

Observed stochastic nodes: 21

Unobserved stochastic nodes: 30

Total graph size: 310

Initializing model

|++++++++++++++++++++++++++++++++++++++++++++++++++| 100%

|**************************************************| 100%

Compiling model graph

Resolving undeclared variables

Allocating nodes

Graph information:

Observed stochastic nodes: 21

Unobserved stochastic nodes: 27

Total graph size: 324

Initializing model

|++++++++++++++++++++++++++++++++++++++++++++++++++| 100%

|**************************************************| 100%

> c<-summary(result.anohe)

> print(c)

Analysis of heterogeneity

=========================

Per-comparison I-squared:

-------------------------

t1 t2 i2.pair i2.cons incons.p

1 Desvenlafaxine100 Desvenlafaxine50 0 0 NA

2 Desvenlafaxine100 Placebo 0 0 NA

3 Desvenlafaxine200 Desvenlafaxine400 NA NA NA

4 Desvenlafaxine200 Placebo NA NA NA

5 Desvenlafaxine25 Desvenlafaxine50 NA NA NA

6 Desvenlafaxine25 Placebo NA NA NA

7 Desvenlafaxine400 Placebo NA NA NA

8 Desvenlafaxine50 Placebo 0 0 NA

Global I-squared:

-------------------------

i2.pair i2.cons

1 0 0


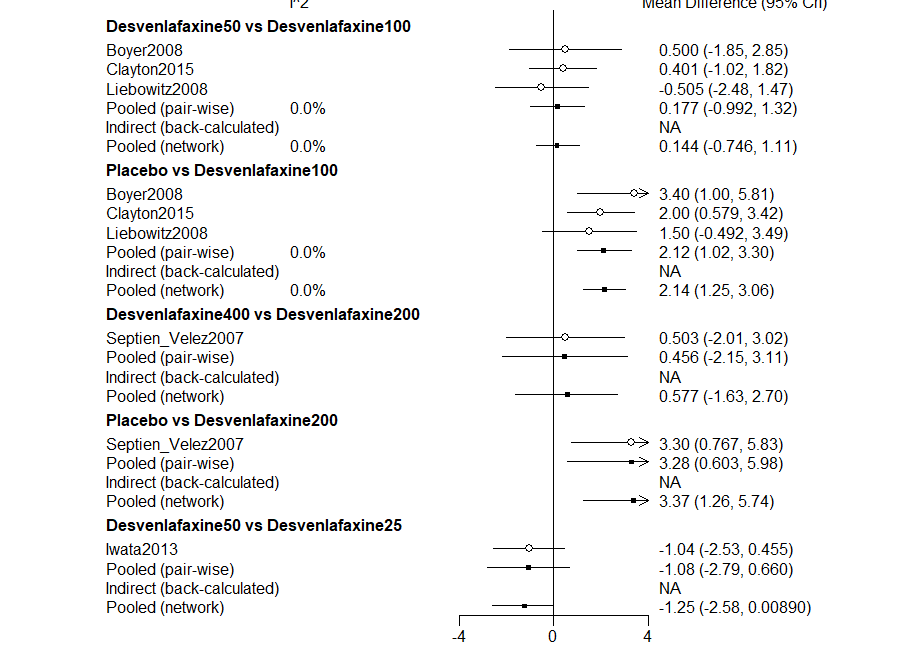


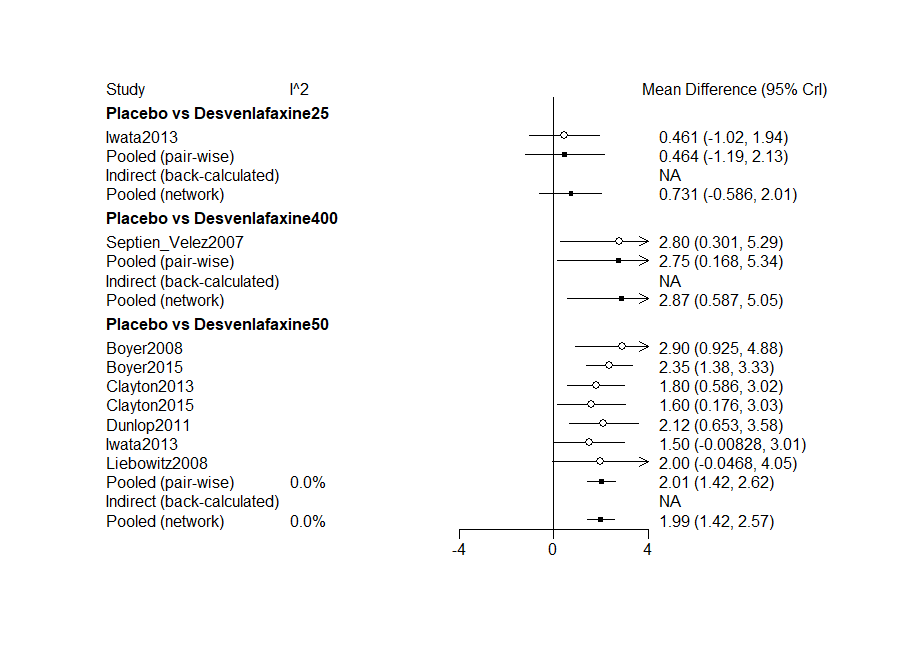


## Publication Bias

> metabias(m.netmeta,order=(ord),pooled='random',method.bias='Egger')

Linear regression test of funnel plot asymmetry

Test result: t = -0.96, df = 16, p-value = 0.3509

Bias estimate: -0.5979 (SE = 0.6222)

Details:

- multiplicative residual heterogeneity variance (tau^2 = 0.3594)

- predictor: standard error

- weight: inverse variance

- reference: Egger et al. (1997), BMJ


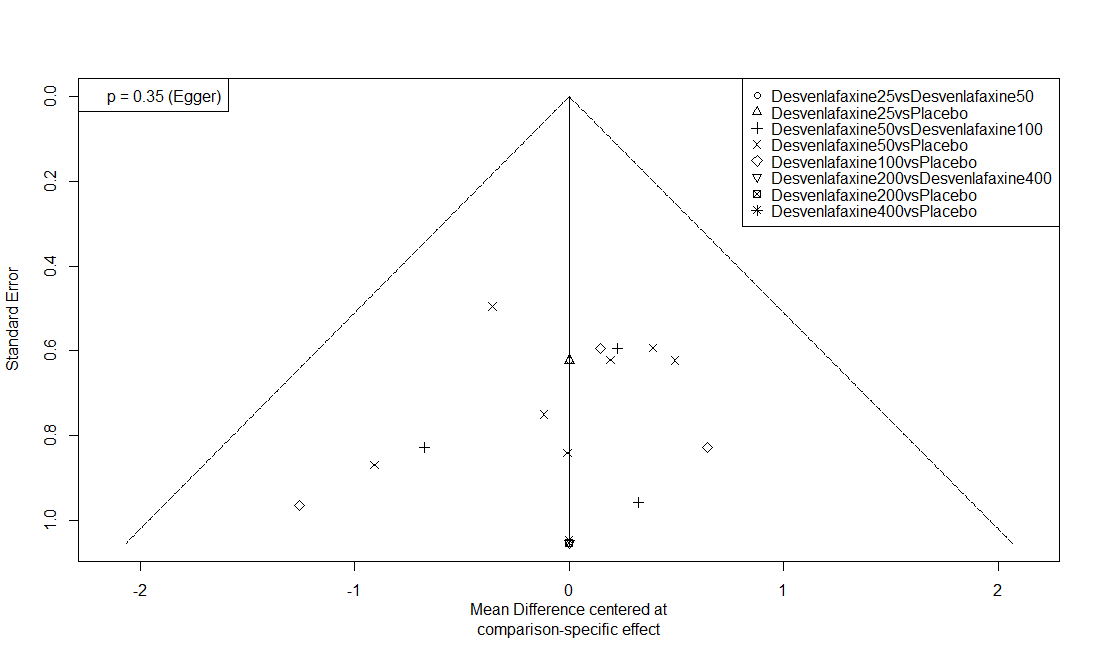


## Density and Trajectory Plots


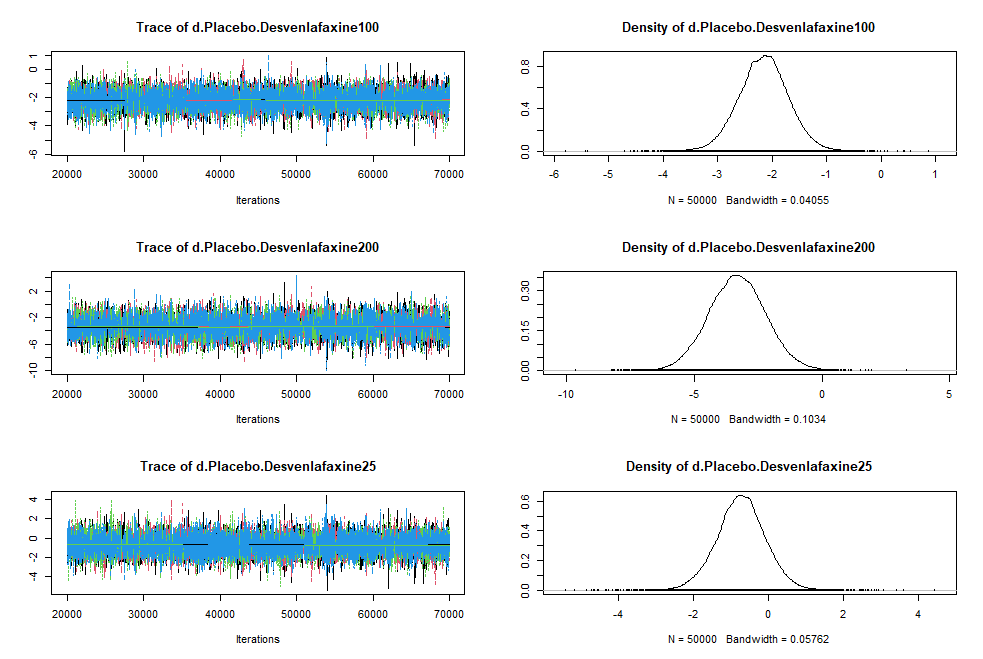

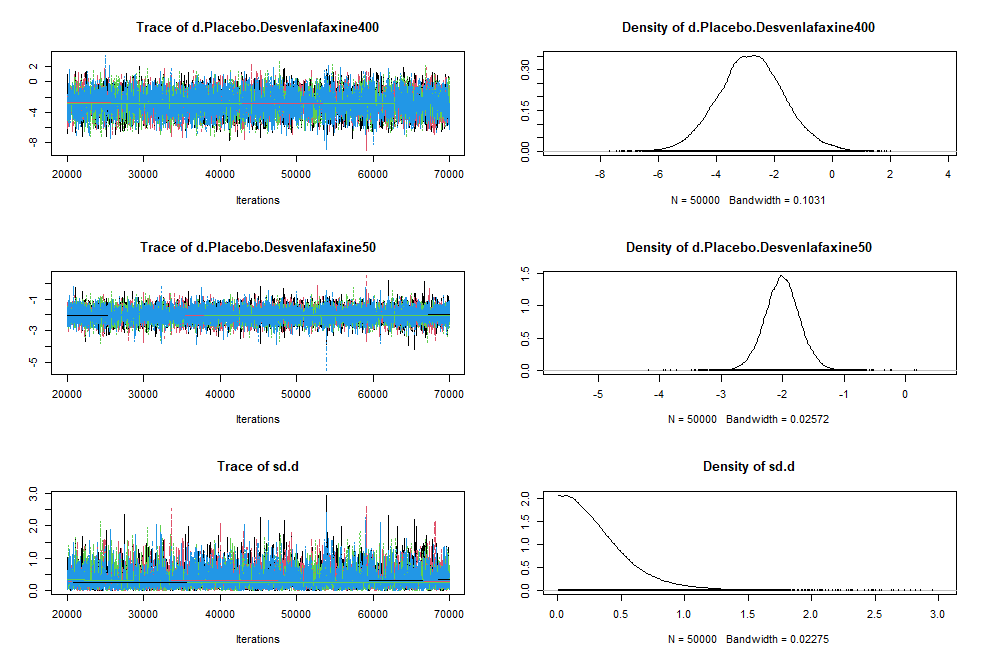


## Convergence Diagnostic Plots


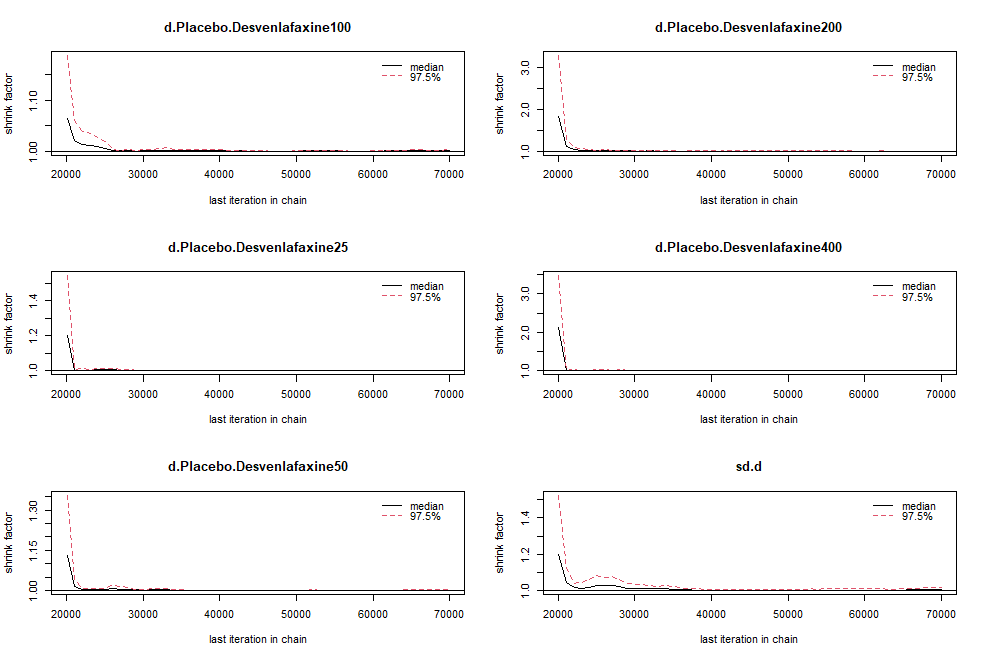


# CGI-S

## Inconsistency Modeling

> modelume<-mtc.model(network,type="ume",n.chain=4,likelihood="normal",link="identity",linearModel="random")

> resultsume <- mtc.run(modelume, n.adapt = 20000, n.iter = 50000, thin = 1)

Compiling model graph

Resolving undeclared variables

Allocating nodes

Graph information:

Observed stochastic nodes: 16

Unobserved stochastic nodes: 24

Total graph size: 229

Initializing model

|++++++++++++++++++++++++++++++++++++++++++++++++++| 100%

|**************************************************| 100%

> summary(resultsume)

Results on the Mean Difference scale

Iterations = 20001:70000

Thinning interval = 1

Number of chains = 4

Sample size per chain = 50000

1. Empirical mean and standard deviation for each variable,

plus standard error of the mean:

Mean SD Naive SE Time-series SE

d.Desvenlafaxine100.Desvenlafaxine50 -0.01876 0.1894 0.0004235 0.0014756

d.Desvenlafaxine100.Placebo 0.39899 0.1843 0.0004121 0.0013713

d.Desvenlafaxine200.Desvenlafaxine400 0.20104 0.2678 0.0005987 0.0020728

d.Desvenlafaxine200.Placebo 0.60094 0.2603 0.0005820 0.0018656

d.Desvenlafaxine25.Desvenlafaxine50 -0.02207 0.2284 0.0005107 0.0010970

d.Desvenlafaxine25.Placebo 0.18845 0.2285 0.0005110 0.0011103

d.Desvenlafaxine50.Placebo 0.42148 0.1586 0.0003547 0.0005854

sd.d 0.15660 0.1326 0.0002966 0.0020196

2. Quantiles for each variable:

2.5% 25% 50% 75% 97.5%

d.Desvenlafaxine100.Desvenlafaxine50 -0.390987 -0.12955 -0.02210 0.08960 0.3706

d.Desvenlafaxine100.Placebo 0.026761 0.29414 0.39968 0.50409 0.7715

d.Desvenlafaxine200.Desvenlafaxine400 -0.335758 0.04747 0.19992 0.35507 0.7396

d.Desvenlafaxine200.Placebo 0.073680 0.45667 0.59800 0.74640 1.1320

d.Desvenlafaxine25.Desvenlafaxine50 -0.501042 -0.13393 -0.02316 0.08879 0.4592

d.Desvenlafaxine25.Placebo -0.288567 0.07838 0.18674 0.29881 0.6695

d.Desvenlafaxine50.Placebo 0.083987 0.34702 0.42256 0.49667 0.7544

sd.d 0.005194 0.05421 0.11971 0.22301 0.5052

-- Model fit (residual deviance):

Dbar pD DIC

15.75247 14.59743 30.34990

16 data points, ratio 0.9845, I^2 = 5%

## Consistency Modeling

model<-mtc.model(network,type="consistency",n.chain=4,likelihood="normal",link="identity",linearModel="random")

> results <- mtc.run(model, n.adapt = 20000, n.iter = 50000, thin = 1)

Compiling model graph

Resolving undeclared variables

Allocating nodes

Graph information:

Observed stochastic nodes: 16

Unobserved stochastic nodes: 22

Total graph size: 263

Initializing model

|++++++++++++++++++++++++++++++++++++++++++++++++++| 100%

|**************************************************| 100%

> summary(results)

Results on the Mean Difference scale

Iterations = 20001:70000

Thinning interval = 1

Number of chains = 4

Sample size per chain = 50000

1. Empirical mean and standard deviation for each variable,

plus standard error of the mean:

Mean SD Naive SE Time-series SE

d.Placebo.Desvenlafaxine100 -0.3808 0.14776 0.0003304 0.0011975

d.Placebo.Desvenlafaxine200 -0.6040 0.22235 0.0004972 0.0018724

d.Placebo.Desvenlafaxine25 -0.2704 0.16590 0.0003710 0.0008615

d.Placebo.Desvenlafaxine400 -0.3989 0.21425 0.0004791 0.0013601

d.Placebo.Desvenlafaxine50 -0.3720 0.08645 0.0001933 0.0004244

sd.d 0.1239 0.09621 0.0002151 0.0012701

2. Quantiles for each variable:

2.5% 25% 50% 75% 97.5%

d.Placebo.Desvenlafaxine100 -0.673601 -0.47151 -0.3811 -0.2903 -0.08982

d.Placebo.Desvenlafaxine200 -1.041704 -0.73979 -0.6054 -0.4685 -0.16296

d.Placebo.Desvenlafaxine25 -0.605519 -0.36098 -0.2707 -0.1796 0.06422

d.Placebo.Desvenlafaxine400 -0.824731 -0.52812 -0.3978 -0.2702 0.02498

d.Placebo.Desvenlafaxine50 -0.545435 -0.41975 -0.3722 -0.3244 -0.19770

sd.d 0.006345 0.05415 0.1029 0.1685 0.37759

-- Model fit (residual deviance):

Dbar pD DIC

15.84619 13.54083 29.38703

16 data points, ratio 0.9904, I^2 = 5%

## Heterogeneity Analysis

> result.anohe <- mtc.anohe(network)

Compiling model graph

Resolving undeclared variables

Allocating nodes

Graph information:

Observed stochastic nodes: 16

Unobserved stochastic nodes: 16

Total graph size: 164

Initializing model

|**************************************************| 100%

Compiling model graph

Resolving undeclared variables

Allocating nodes

Graph information:

Observed stochastic nodes: 16

Unobserved stochastic nodes: 25

Total graph size: 244

Initializing model

|++++++++++++++++++++++++++++++++++++++++++++++++++| 100%

|**************************************************| 100%

Compiling model graph

Resolving undeclared variables

Allocating nodes

Graph information:

Observed stochastic nodes: 16

Unobserved stochastic nodes: 22

Total graph size: 263

Initializing model

|++++++++++++++++++++++++++++++++++++++++++++++++++| 100%

|**************************************************| 100%

> c<-summary(result.anohe)

> print(c)

Analysis of heterogeneity

=========================

Per-comparison I-squared:

-------------------------

t1 t2 i2.pair i2.cons incons.p

1 Desvenlafaxine100 Desvenlafaxine50 0.00000 0.00000 NA

2 Desvenlafaxine100 Placebo 53.14032 54.26102 NA

3 Desvenlafaxine200 Desvenlafaxine400 NA NA NA

4 Desvenlafaxine200 Placebo NA NA NA

5 Desvenlafaxine25 Desvenlafaxine50 NA NA NA

6 Desvenlafaxine25 Placebo NA NA NA

7 Desvenlafaxine400 Placebo NA NA NA

8 Desvenlafaxine50 Placebo 0.00000 0.00000 NA

Global I-squared:

-------------------------

i2.pair i2.cons

1 0 0


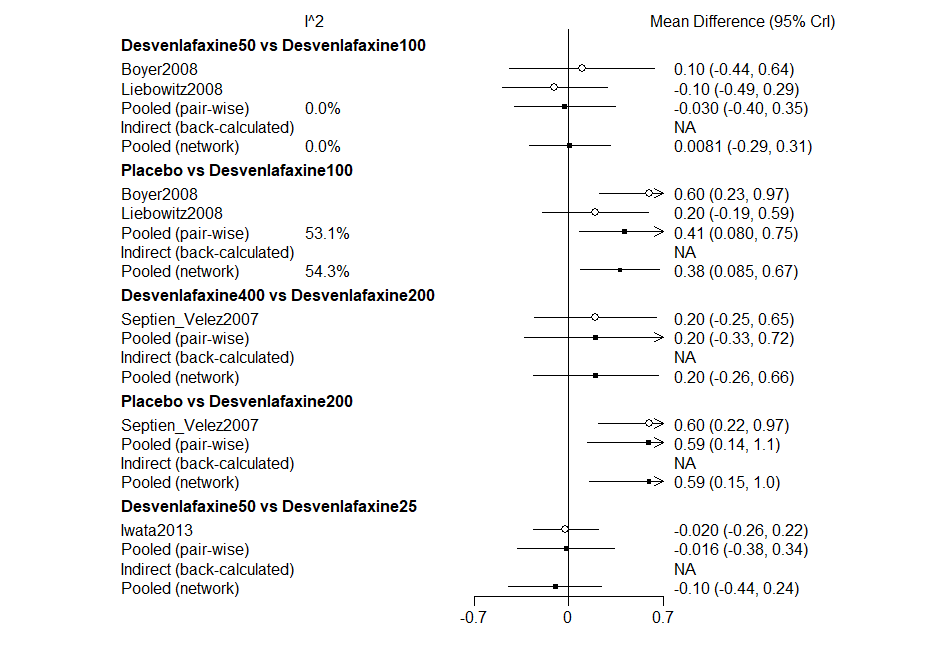


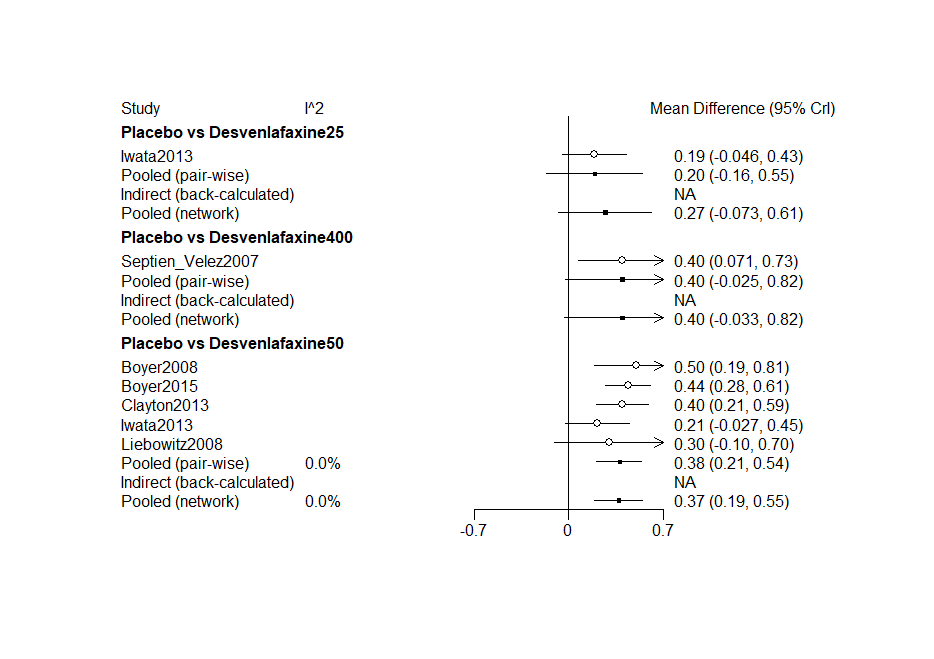


## Publication Bias

> metabias(m.netmeta,order=(ord),pooled='random',method.bias='Egger')

Linear regression test of funnel plot asymmetry

Test result: t = 0.12, df = 12, p-value = 0.9037

Bias estimate: 0.1024 (SE = 0.8289)

Details:

- multiplicative residual heterogeneity variance (tau^2 = 0.6676)

- predictor: standard error

- weight: inverse variance

- reference: Egger et al. (1997), BMJ


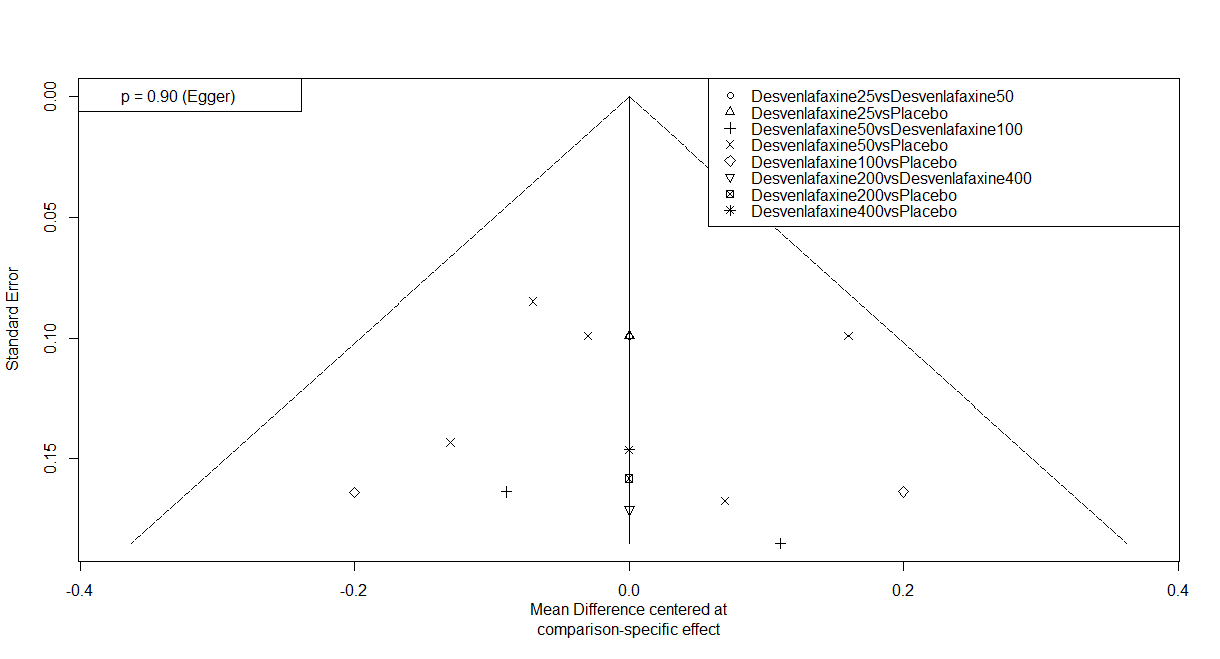


## Density and Trajectory Plots


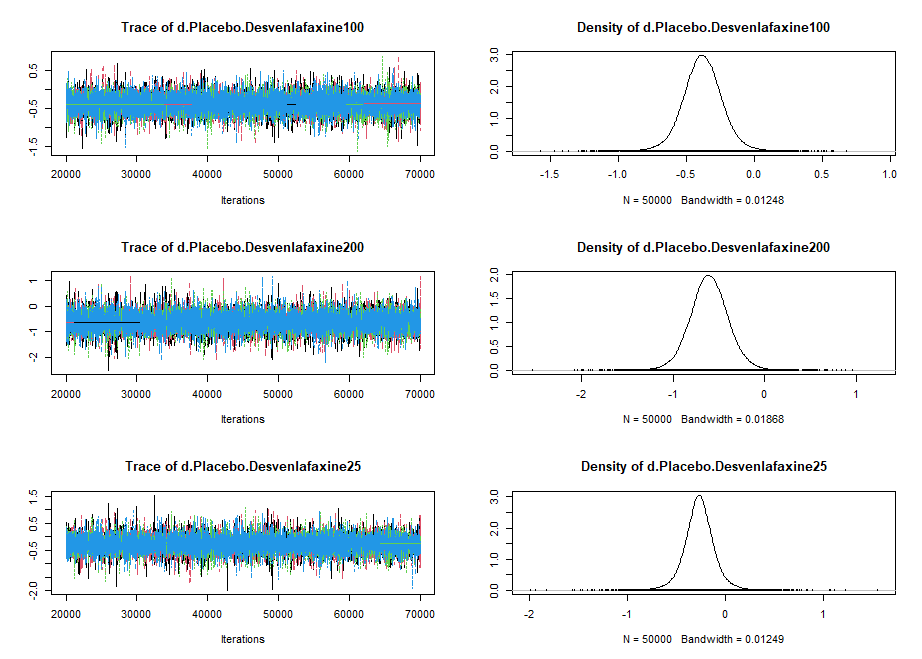


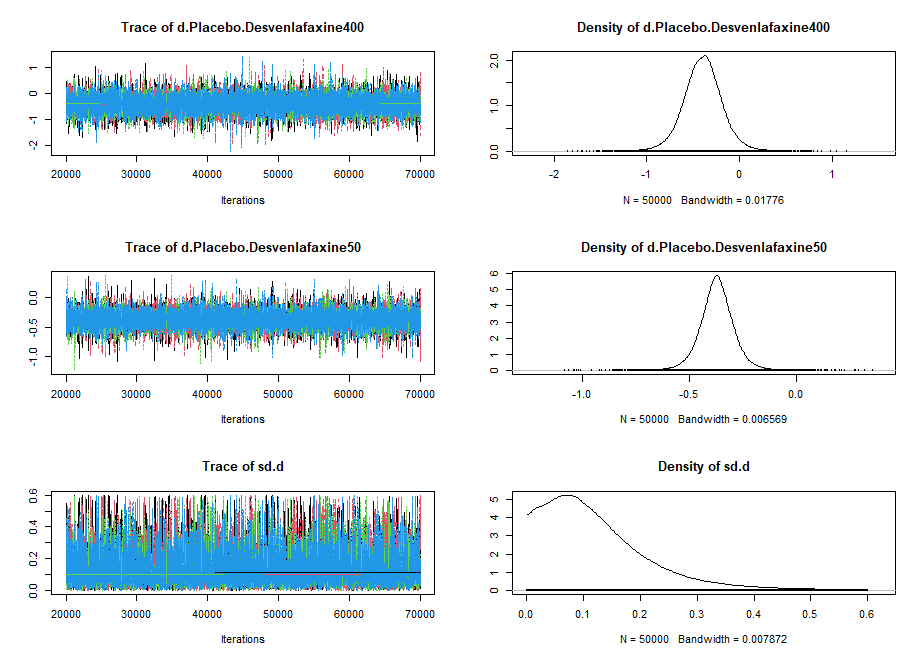


## Convergence Diagnostic Plots


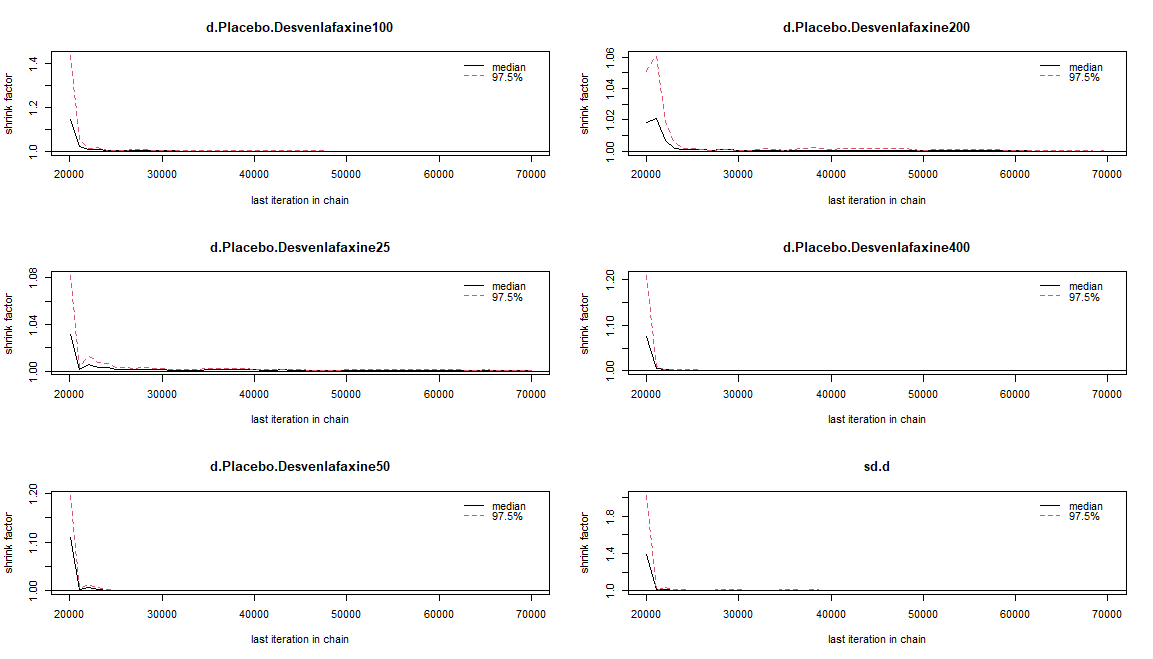


# MADRS

## Inconsistency Modeling

> modelume<-mtc.model(network,type="ume",n.chain=4,likelihood="normal",link="identity",linearModel="random")

> resultsume <- mtc.run(modelume, n.adapt = 20000, n.iter = 50000, thin = 1)

Compiling model graph

Resolving undeclared variables

Allocating nodes

Graph information:

Observed stochastic nodes: 14

Unobserved stochastic nodes: 22

Total graph size: 207

Initializing model

|++++++++++++++++++++++++++++++++++++++++++++++++++| 100%

|**************************************************| 100%

> summary(resultsume)

Results on the Mean Difference scale

Iterations = 20001:70000

Thinning interval = 1

Number of chains = 4

Sample size per chain = 50000

1. Empirical mean and standard deviation for each variable,

plus standard error of the mean:

Mean SD Naive SE Time-series SE

d.Desvenlafaxine100.Desvenlafaxine50 0.1447 1.554 0.003474 0.009593

d.Desvenlafaxine100.Placebo 3.0715 1.548 0.003460 0.009301

d.Desvenlafaxine200.Desvenlafaxine400 0.6953 2.231 0.004988 0.014065

d.Desvenlafaxine200.Placebo 4.2900 2.149 0.004805 0.012882

d.Desvenlafaxine25.Desvenlafaxine50 -1.0661 2.011 0.004496 0.008852

d.Desvenlafaxine25.Placebo 0.9977 2.016 0.004507 0.008914

d.Desvenlafaxine50.Placebo 2.6915 2.053 0.004591 0.008167

sd.d 1.4574 1.096 0.002450 0.015130

2. Quantiles for each variable:

2.5% 25% 50% 75% 97.5%

d.Desvenlafaxine100.Desvenlafaxine50 -3.06503 -0.71567 0.1294 0.99949 3.390

d.Desvenlafaxine100.Placebo -0.11836 2.21512 3.0644 3.92572 6.307

d.Desvenlafaxine200.Desvenlafaxine400 -3.87294 -0.56425 0.6982 1.95903 5.264

d.Desvenlafaxine200.Placebo -0.17290 3.10290 4.2869 5.45574 8.772

d.Desvenlafaxine25.Desvenlafaxine50 -5.32703 -2.07315 -1.0712 -0.06906 3.256

d.Desvenlafaxine25.Placebo -3.24887 -0.01819 0.9884 2.00367 5.306

d.Desvenlafaxine50.Placebo -1.63311 1.62764 2.6860 3.74943 7.041

sd.d 0.05818 0.55551 1.2054 2.15807 3.960

-- Model fit (residual deviance):

Dbar pD DIC

13.64819 13.13325 26.78143

14 data points, ratio 0.9749, I^2 = 5%

## Consistency Modeling

> model<-mtc.model(network,type="consistency",n.chain=4,likelihood="normal",link="identity",linearModel="random")

> results <- mtc.run(model, n.adapt = 20000, n.iter = 50000, thin = 1)

Compiling model graph

Resolving undeclared variables

Allocating nodes

Graph information:

Observed stochastic nodes: 14

Unobserved stochastic nodes: 20

Total graph size: 241

Initializing model

|++++++++++++++++++++++++++++++++++++++++++++++++++| 100%

|**************************************************| 100%

> summary(results)

Results on the Mean Difference scale

Iterations = 20001:70000

Thinning interval = 1

Number of chains = 4

Sample size per chain = 50000

1. Empirical mean and standard deviation for each variable,

plus standard error of the mean:

Mean SD Naive SE Time-series SE

d.Placebo.Desvenlafaxine100 -2.8786 1.0885 0.002434 0.011147

d.Placebo.Desvenlafaxine200 -4.2999 1.5686 0.003507 0.015120

d.Placebo.Desvenlafaxine25 -1.2897 1.2494 0.002794 0.008614

d.Placebo.Desvenlafaxine400 -3.5995 1.5632 0.003495 0.012568

d.Placebo.Desvenlafaxine50 -2.6086 0.7355 0.001645 0.005277

sd.d 0.8066 0.7372 0.001648 0.011112

2. Quantiles for each variable:

2.5% 25% 50% 75% 97.5%

d.Placebo.Desvenlafaxine100 -5.03860 -3.5305 -2.8696 -2.2186 -0.7721

d.Placebo.Desvenlafaxine200 -7.37325 -5.2361 -4.3088 -3.3649 -1.1824

d.Placebo.Desvenlafaxine25 -3.79596 -1.9760 -1.2815 -0.5969 1.1787

d.Placebo.Desvenlafaxine400 -6.67689 -4.5291 -3.6075 -2.6704 -0.5218

d.Placebo.Desvenlafaxine50 -4.08318 -3.0285 -2.6008 -2.1786 -1.1757

sd.d 0.02421 0.2683 0.6019 1.1118 2.8569

-- Model fit (residual deviance):

Dbar pD DIC

12.58065 11.38245 23.96310

14 data points, ratio 0.8986, I^2 = 0%

## Heterogeneity Analysis

> result.anohe <- mtc.anohe(network)

Compiling model graph

Resolving undeclared variables

Allocating nodes

Graph information:

Observed stochastic nodes: 14

Unobserved stochastic nodes: 14

Total graph size: 145

Initializing model

|**************************************************| 100%

Compiling model graph

Resolving undeclared variables

Allocating nodes

Graph information:

Observed stochastic nodes: 14

Unobserved stochastic nodes: 23

Total graph size: 220

Initializing model

|++++++++++++++++++++++++++++++++++++++++++++++++++| 100%

|**************************************************| 100%

Compiling model graph

Resolving undeclared variables

Allocating nodes

Graph information:

Observed stochastic nodes: 14

Unobserved stochastic nodes: 20

Total graph size: 241

Initializing model

|++++++++++++++++++++++++++++++++++++++++++++++++++| 100%

|**************************************************| 100%

> c<-summary(result.anohe)

> print(c)

Analysis of heterogeneity

=========================

Per-comparison I-squared:

-------------------------

t1 t2 i2.pair i2.cons incons.p

1 Desvenlafaxine100 Desvenlafaxine50 0.000000 0.0000 NA

2 Desvenlafaxine100 Placebo 7.483479 9.1676 NA

3 Desvenlafaxine200 Desvenlafaxine400 NA NA NA

4 Desvenlafaxine200 Placebo NA NA NA

5 Desvenlafaxine25 Desvenlafaxine50 NA NA NA

6 Desvenlafaxine25 Placebo NA NA NA

7 Desvenlafaxine400 Placebo NA NA NA

8 Desvenlafaxine50 Placebo 0.000000 0.0000 NA

Global I-squared:

-------------------------

i2.pair i2.cons

1 0 0


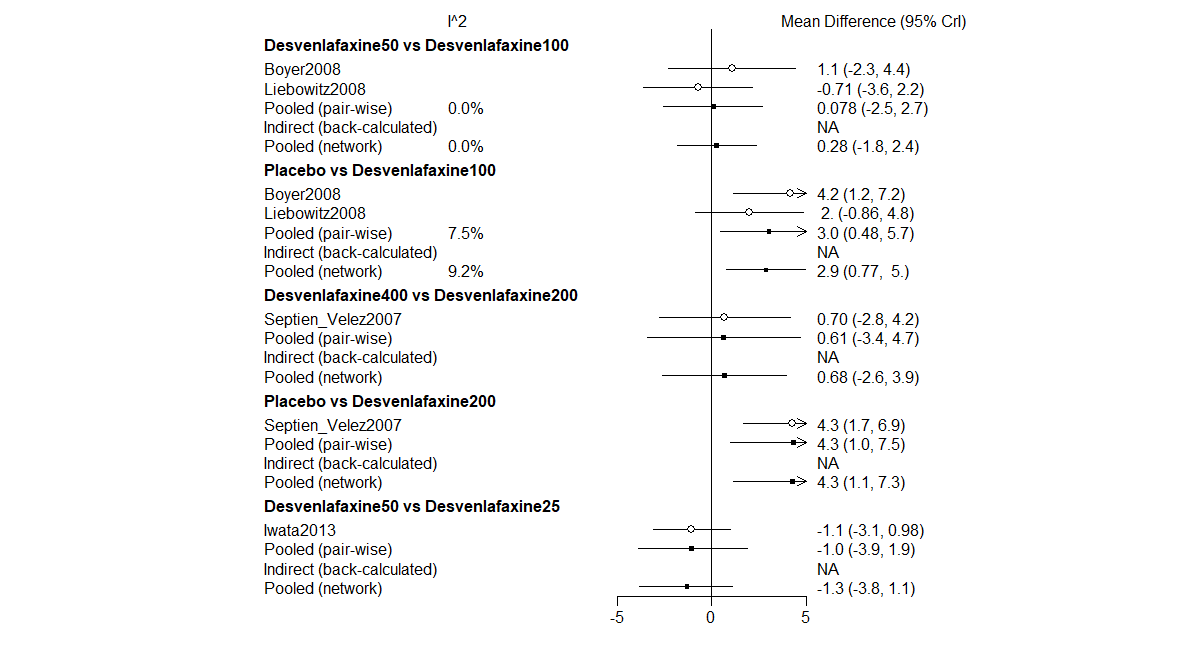


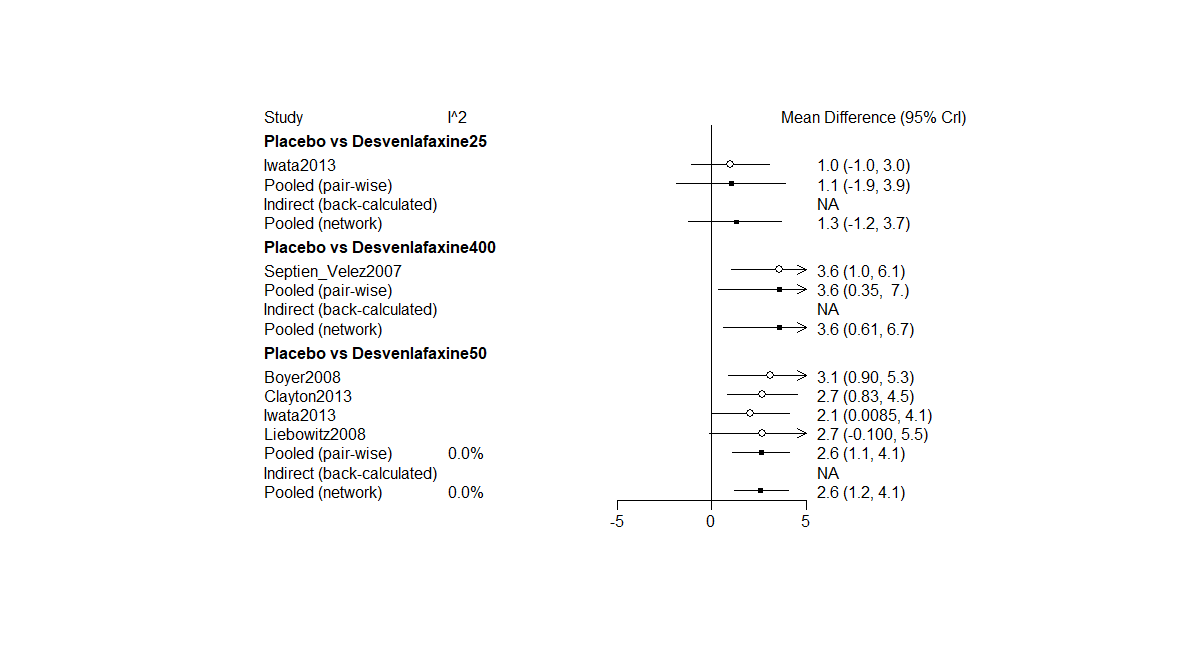


## Publication Bias

> metabias(m.netmeta,order=(ord),pooled='random',method.bias='Egger')

Linear regression test of funnel plot asymmetry

Test result: t = -0.33, df = 11, p-value = 0.7507

Bias estimate: -0.3236 (SE = 0.9934)

Details:

- multiplicative residual heterogeneity variance (tau^2 = 0.3035)

- predictor: standard error

- weight: inverse variance

- reference: Egger et al. (1997), BMJ


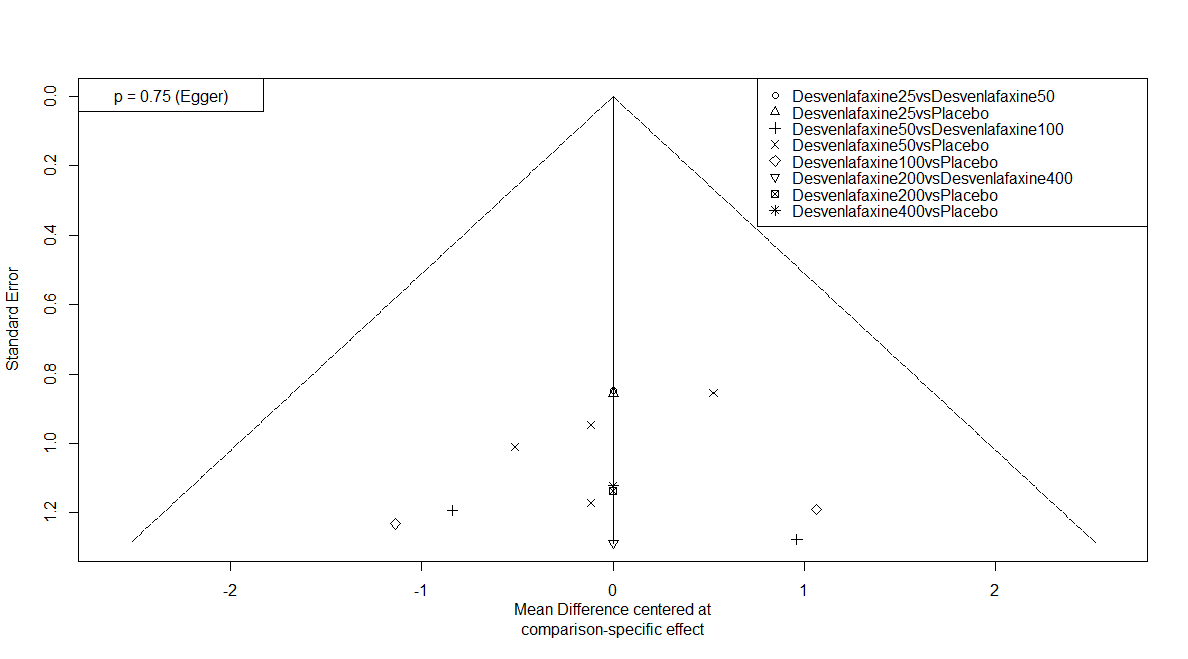


## Density and Trajectory Plots


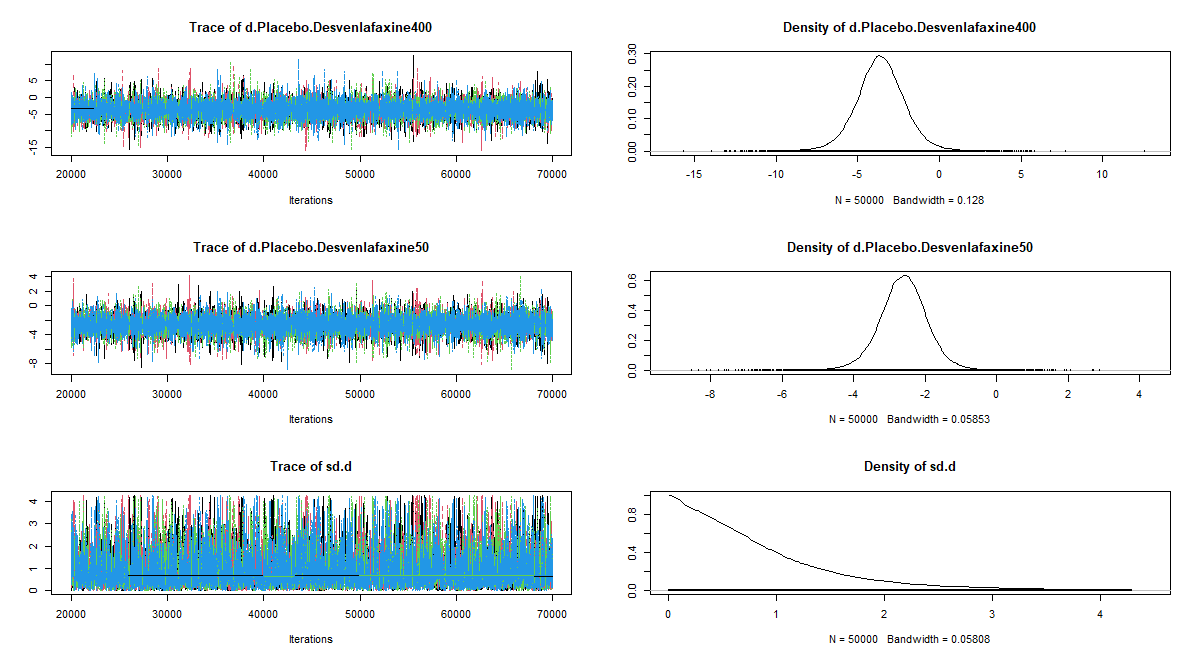


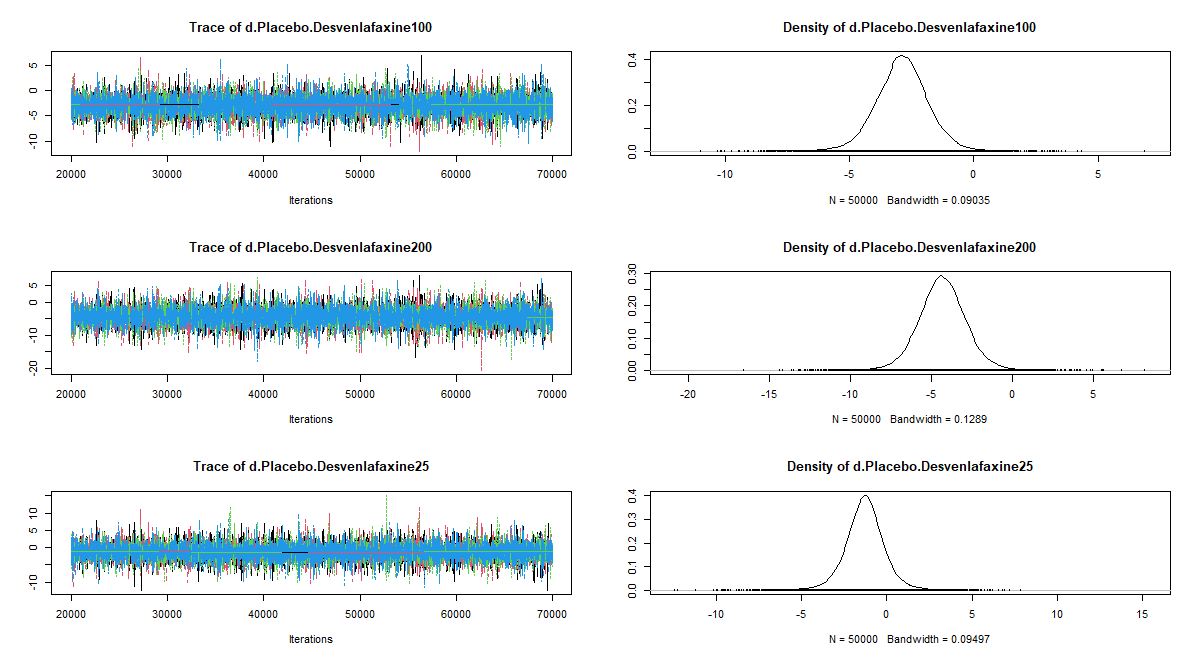


## Convergence Diagnostic Plots


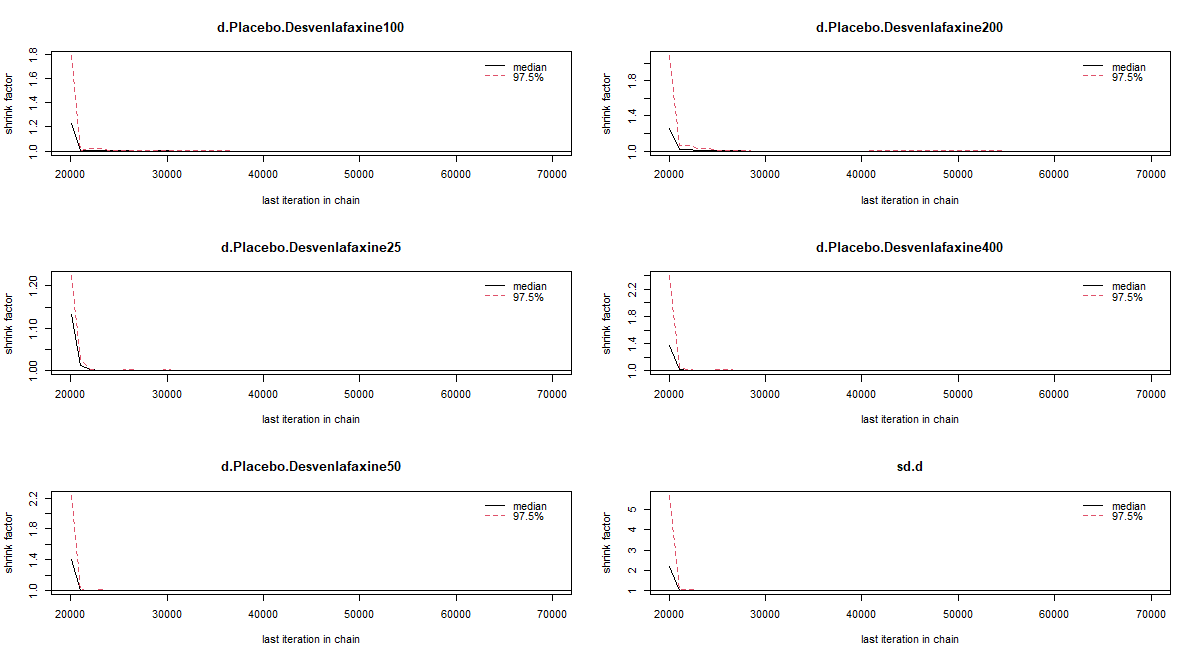


# TEAEs

## Inconsistency Modeling

> modelume<-mtc.model(network,type="ume",n.chain=4,likelihood="binom",link="log",linearModel="random")

> resultsume <- mtc.run(modelume, n.adapt = 20000, n.iter = 50000, thin = 1)

Compiling model graph

Resolving undeclared variables

Allocating nodes

Graph information:

Observed stochastic nodes: 19

Unobserved stochastic nodes: 27

Total graph size: 464

Initializing model

|++++++++++++++++++++++++++++++++++++++++++++++++++| 100%

|**************************************************| 100%

> summary(resultsume)

Results on the Log Risk Ratio scale

Iterations = 20001:70000

Thinning interval = 1

Number of chains = 4

Sample size per chain = 50000

1. Empirical mean and standard deviation for each variable,

plus standard error of the mean:

Mean SD Naive SE Time-series SE

d.Desvenlafaxine100.Desvenlafaxine50 0.00705 0.2119 0.0004739 0.0006376

d.Desvenlafaxine100.Placebo -0.34033 0.2147 0.0004800 0.0006652

d.Desvenlafaxine200.Desvenlafaxine400 0.06850 0.3641 0.0008142 0.0010974

d.Desvenlafaxine200.Placebo -0.18241 0.3682 0.0008233 0.0011089

d.Desvenlafaxine25.Desvenlafaxine50 0.07900 0.3654 0.0008171 0.0011182

d.Desvenlafaxine25.Placebo -0.06693 0.3673 0.0008213 0.0011285

d.Desvenlafaxine50.Placebo -0.10674 0.2594 0.0005801 0.0006206

sd.d 0.33360 0.1404 0.0003139 0.0010174

2. Quantiles for each variable:

2.5% 25% 50% 75% 97.5%

d.Desvenlafaxine100.Desvenlafaxine50 -0.4292 -0.1116 0.007065 0.12550 0.44449

d.Desvenlafaxine100.Placebo -0.7898 -0.4602 -0.334984 -0.21693 0.08827

d.Desvenlafaxine200.Desvenlafaxine400 -0.6793 -0.1351 0.067013 0.27189 0.81981

d.Desvenlafaxine200.Placebo -0.9367 -0.3877 -0.182195 0.02226 0.57911

d.Desvenlafaxine25.Desvenlafaxine50 -0.6743 -0.1251 0.079311 0.28508 0.83029

d.Desvenlafaxine25.Placebo -0.8236 -0.2734 -0.066608 0.14015 0.68761

d.Desvenlafaxine50.Placebo -0.6373 -0.2520 -0.106626 0.03818 0.42541

sd.d 0.1399 0.2307 0.303742 0.40655 0.69347

-- Model fit (residual deviance):

Dbar pD DIC

19.69145 18.64326 38.33471

19 data points, ratio 1.036, I^2 = 9%

## Consistency Modeling

> model<-mtc.model(network,type="consistency",n.chain=4,likelihood="binom",link="log",linearModel="random")

> results <- mtc.run(model, n.adapt = 20000, n.iter = 50000, thin = 1)

Compiling model graph

Resolving undeclared variables

Allocating nodes

Graph information:

Observed stochastic nodes: 19

Unobserved stochastic nodes: 25

Total graph size: 498

Initializing model

|++++++++++++++++++++++++++++++++++++++++++++++++++| 100%

|**************************************************| 100%

> summary(results)

Results on the Log Risk Ratio scale

Iterations = 20001:70000

Thinning interval = 1

Number of chains = 4

Sample size per chain = 50000

1. Empirical mean and standard deviation for each variable,

plus standard error of the mean:

Mean SD Naive SE Time-series SE

d.Placebo.Desvenlafaxine100 0.2754 0.1672 0.0003739 0.0005028

d.Placebo.Desvenlafaxine200 0.1840 0.3052 0.0006824 0.0009696

d.Placebo.Desvenlafaxine25 0.1069 0.2732 0.0006109 0.0007749

d.Placebo.Desvenlafaxine400 0.2516 0.3047 0.0006812 0.0009196

d.Placebo.Desvenlafaxine50 0.2285 0.1260 0.0002819 0.0003712

sd.d 0.2766 0.1102 0.0002465 0.0007423

2. Quantiles for each variable:

2.5% 25% 50% 75% 97.5%

d.Placebo.Desvenlafaxine100 -0.05095 0.176394 0.2707 0.3703 0.6255

d.Placebo.Desvenlafaxine200 -0.43223 0.007613 0.1837 0.3602 0.8046

d.Placebo.Desvenlafaxine25 -0.44283 -0.051935 0.1050 0.2634 0.6656

d.Placebo.Desvenlafaxine400 -0.36301 0.076210 0.2511 0.4261 0.8709

d.Placebo.Desvenlafaxine50 -0.01955 0.154069 0.2250 0.3007 0.4910

sd.d 0.12426 0.198914 0.2551 0.3299 0.5575

-- Model fit (residual deviance):

Dbar pD DIC

20.32239 18.47932 38.80171

19 data points, ratio 1.07, I^2 = 11%

## Heterogeneity Analysis

> result.anohe <- mtc.anohe(network)

Compiling model graph

Resolving undeclared variables

Allocating nodes

Graph information:

Observed stochastic nodes: 19

Unobserved stochastic nodes: 19

Total graph size: 366

Initializing model

|++++++++++++++++++++++++++++++++++++++++++++++++++| 100%

|**************************************************| 100%

Compiling model graph

Resolving undeclared variables

Allocating nodes

Graph information:

Observed stochastic nodes: 19

Unobserved stochastic nodes: 28

Total graph size: 324

Initializing model

|++++++++++++++++++++++++++++++++++++++++++++++++++| 100%

|**************************************************| 100%

Compiling model graph

Resolving undeclared variables

Allocating nodes

Graph information:

Observed stochastic nodes: 19

Unobserved stochastic nodes: 25

Total graph size: 475

Initializing model

|++++++++++++++++++++++++++++++++++++++++++++++++++| 100%

|**************************************************| 100%

> c<-summary(result.anohe)

> print(c)

Analysis of heterogeneity

=========================

Per-comparison I-squared:

-------------------------

t1 t2 i2.pair i2.cons incons.p

1 Desvenlafaxine100 Desvenlafaxine50 23.19342 21.70091 NA

2 Desvenlafaxine100 Placebo 81.95105 82.42271 NA

3 Desvenlafaxine200 Desvenlafaxine400 NA NA NA

4 Desvenlafaxine200 Placebo NA NA NA

5 Desvenlafaxine25 Desvenlafaxine50 NA NA NA

6 Desvenlafaxine25 Placebo NA NA NA

7 Desvenlafaxine400 Placebo NA NA NA

8 Desvenlafaxine50 Placebo 71.63369 71.95187 NA

Global I-squared:

-------------------------

i2.pair i2.cons

1 71.25685 62.71442


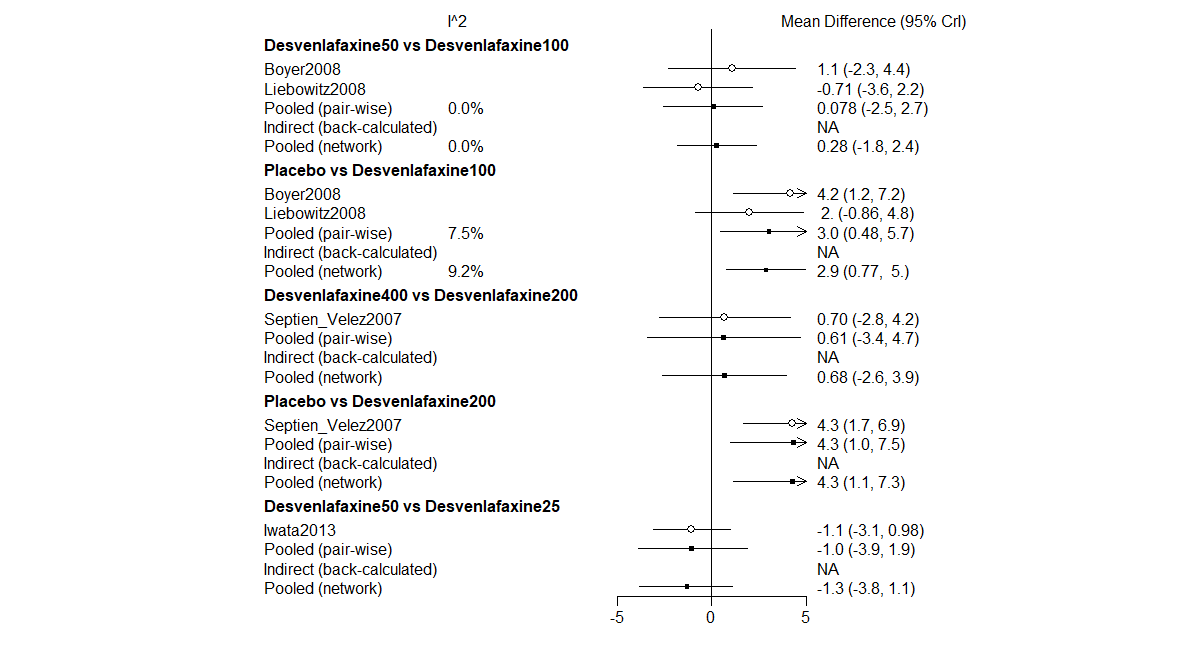


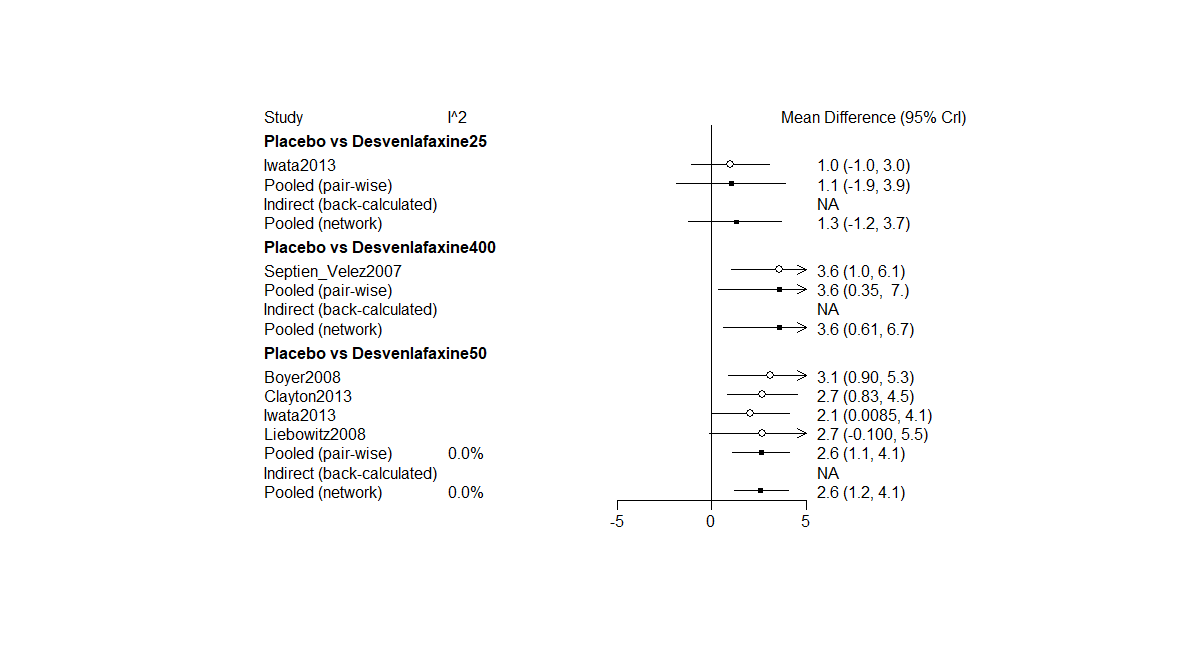


## Publication Bias

Linear regression test of funnel plot asymmetry

Test result: t = 3.22, df = 15, p-value = 0.0058

Bias estimate: 5.2959 (SE = 1.6463)

Details:

- multiplicative residual heterogeneity variance (tau^2 = 2.4183)

- predictor: standard error

- weight: inverse variance

- reference: Egger et al. (1997), BMJ


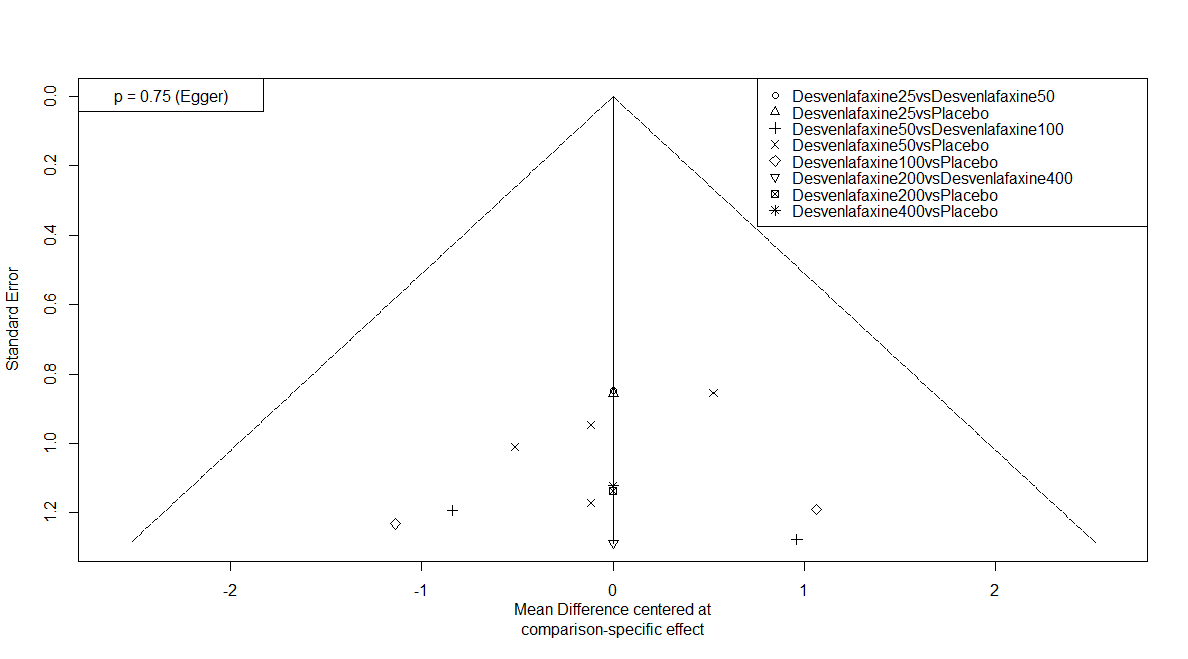


## Density and Trajectory Plots


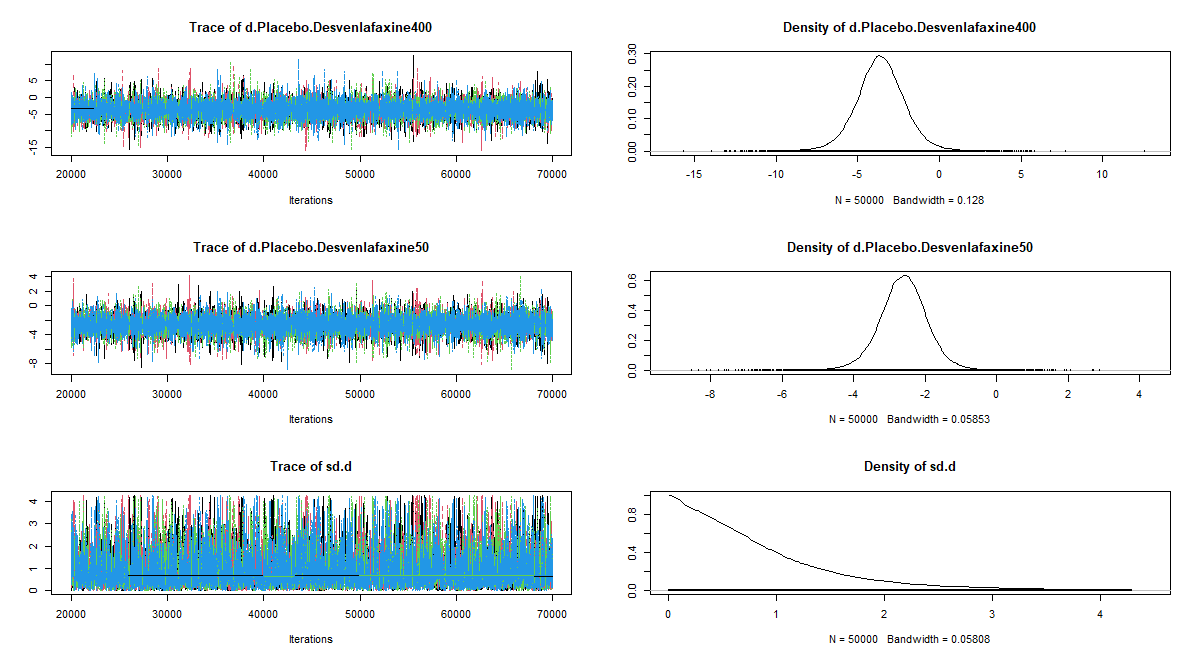


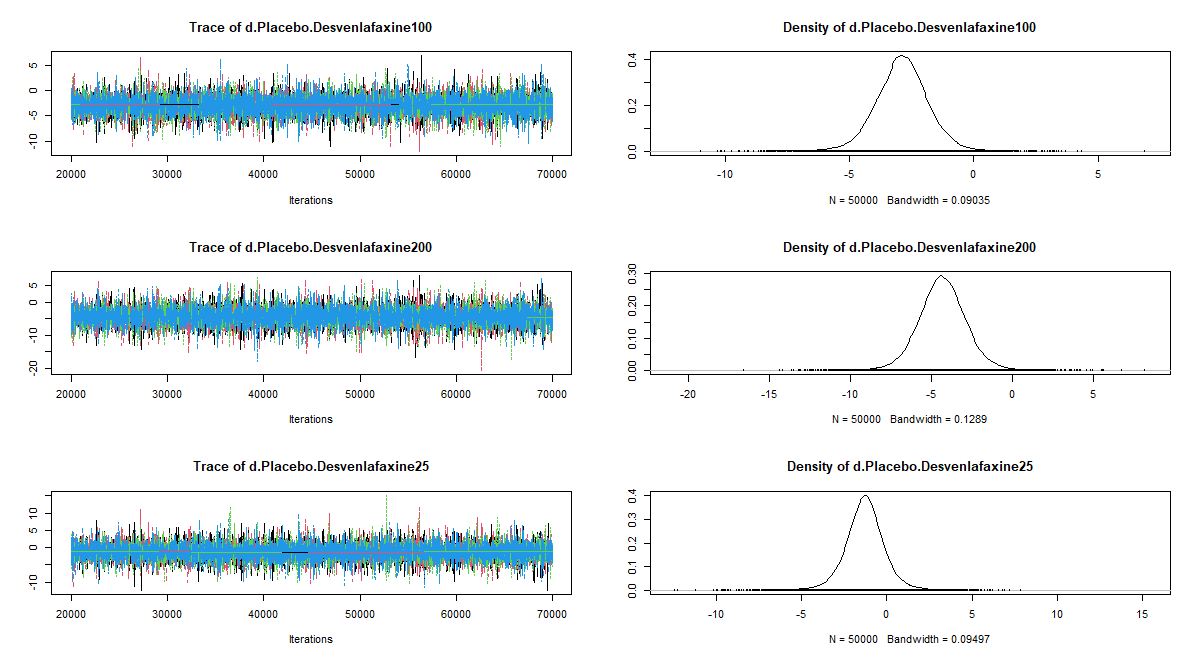


## Convergence Diagnostic Plots


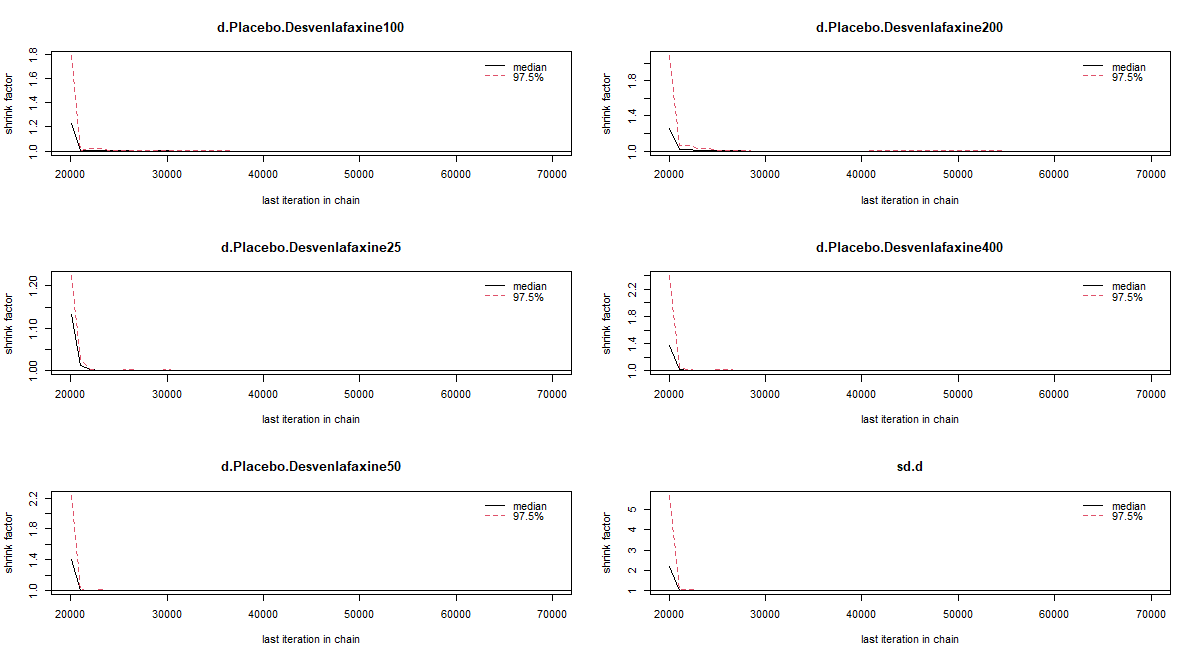

Supplement: Supplementary file 2 [file Data_Sheet_2.docx]
