# Supplementary material for: Efficacy and safety of desvenlafaxine in treating patients with major depressive disorder: a network meta-analysis
Source: Front Neurosci. 2026 Feb 6;20:1721852. doi: 10.3389/fnins.2026.1721852 (PMC12920435; doi:10.3389/fnins.2026.1721852)
Supplement: Supplementary file 3 [file Data_Sheet_3.docx]

# HAM-D17

## Excluding the 25 mg dose

### Sucra Ranking Diagram


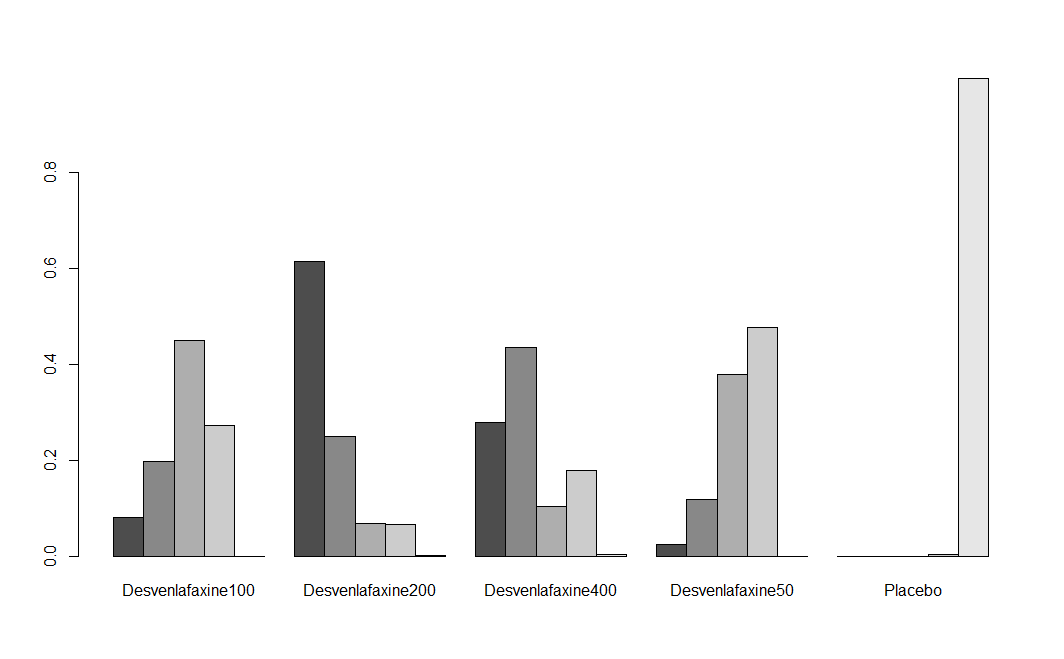


### League Table

|  | Desvenlafaxine100 | Desvenlafaxine200 | Desvenlafaxine400 | Desvenlafaxine50 | Placebo |
| --- | --- | --- | --- | --- | --- |
| Desvenlafaxine100 | Desvenlafaxine100 | -1.15 (-3.35, 1.07) | -0.65 (-2.85, 1.55) | 0.15 (-0.62, 0.93) | 2.14 (1.37, 2.92) |
| Desvenlafaxine200 | 1.15 (-1.07, 3.35) | Desvenlafaxine200 | 0.49 (-1.57, 2.56) | 1.3 (-0.83, 3.41) | 3.29 (1.22, 5.35) |
| Desvenlafaxine400 | 0.65 (-1.55, 2.85) | -0.49 (-2.56, 1.57) | Desvenlafaxine400 | 0.8 (-1.3, 2.91) | 2.79 (0.74, 4.85) |
| Desvenlafaxine50 | -0.15 (-0.93, 0.62) | -1.3 (-3.41, 0.83) | -0.8 (-2.91, 1.3) | Desvenlafaxine50 | 1.99 (1.51, 2.47) |
| Placebo | **-2.14 (-2.92, -1.37)** | **-3.29 (-5.35, -1.22)** | **-2.79 (-4.85, -0.74)** | **-1.99 (-2.47, -1.51)** | Placebo |

## **Restricting to 8-week trials**

### Sucra Ranking Diagram


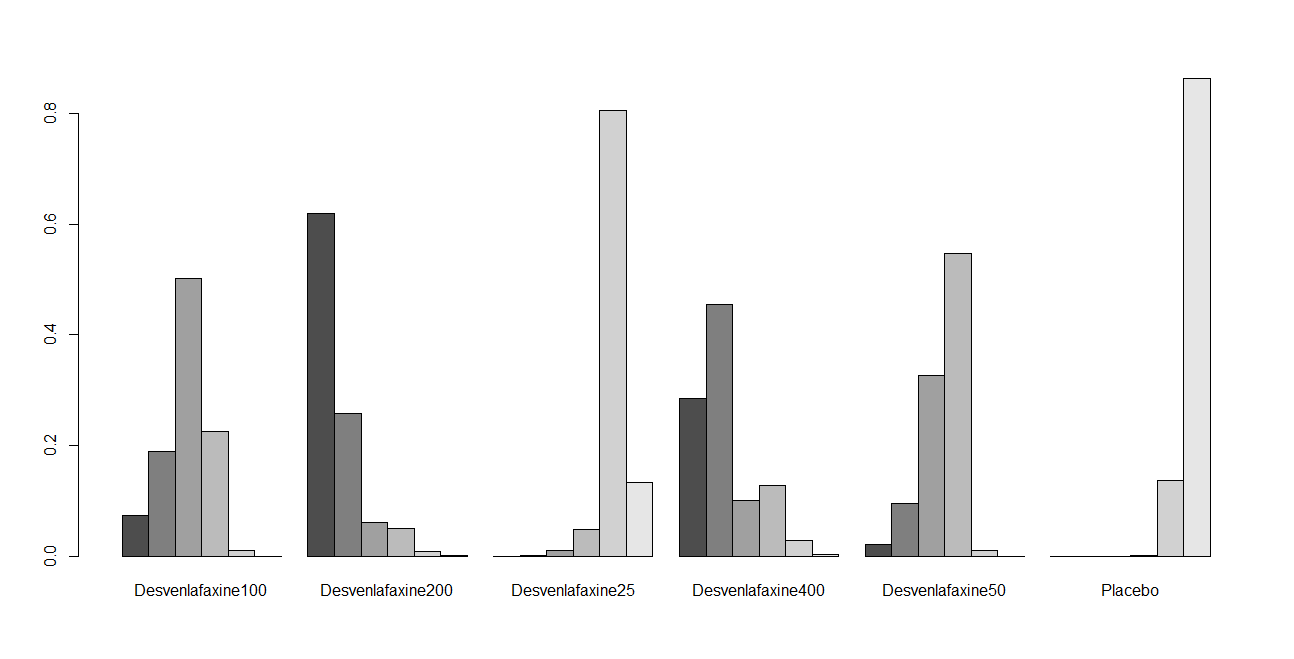


### League Table

|  | Desvenlafaxine100 | Desvenlafaxine200 | Desvenlafaxine25 | Desvenlafaxine400 | Desvenlafaxine50 | Placebo |
| --- | --- | --- | --- | --- | --- | --- |
| Desvenlafaxine100 | Desvenlafaxine100 | -1.22 (-3.45, 1) | 1.44 (0.15, 2.73) | -0.73 (-2.95, 1.48) | 0.23 (-0.59, 1.04) | 2.07 (1.26, 2.89) |
| Desvenlafaxine200 | 1.22 (-1, 3.45) | Desvenlafaxine200 | 2.66 (0.31, 5) | 0.49 (-1.58, 2.57) | 1.44 (-0.74, 3.63) | 3.29 (1.22, 5.35) |
| Desvenlafaxine25 | **-1.44 (-2.73, -0.15)** | **-2.66 (-5, -0.31)** | Desvenlafaxine25 | -2.17 (-4.51, 0.17) | -1.22 (-2.33, -0.11) | 0.63 (-0.48, 1.74) |
| Desvenlafaxine400 | 0.73 (-1.48, 2.95) | -0.49 (-2.57, 1.58) | 2.17 (-0.17, 4.51) | Desvenlafaxine400 | 0.95 (-1.21, 3.12) | 2.8 (0.75, 4.85) |
| Desvenlafaxine50 | -0.23 (-1.04, 0.59) | -1.44 (-3.63, 0.74) | **1.22 (0.11, 2.33)** | -0.95 (-3.12, 1.21) | Desvenlafaxine50 | 1.85 (1.16, 2.53) |
| Placebo | **-2.07 (-2.89, -1.26)** | **-3.29 (-5.35, -1.22)** | -0.63 (-1.74, 0.48) | **-2.8 (-4.85, -0.75)** | **-1.85 (-2.53, -1.16)** | Placebo |

# CGI-S

## Excluding the 25 mg dose

### Sucra Ranking Diagram


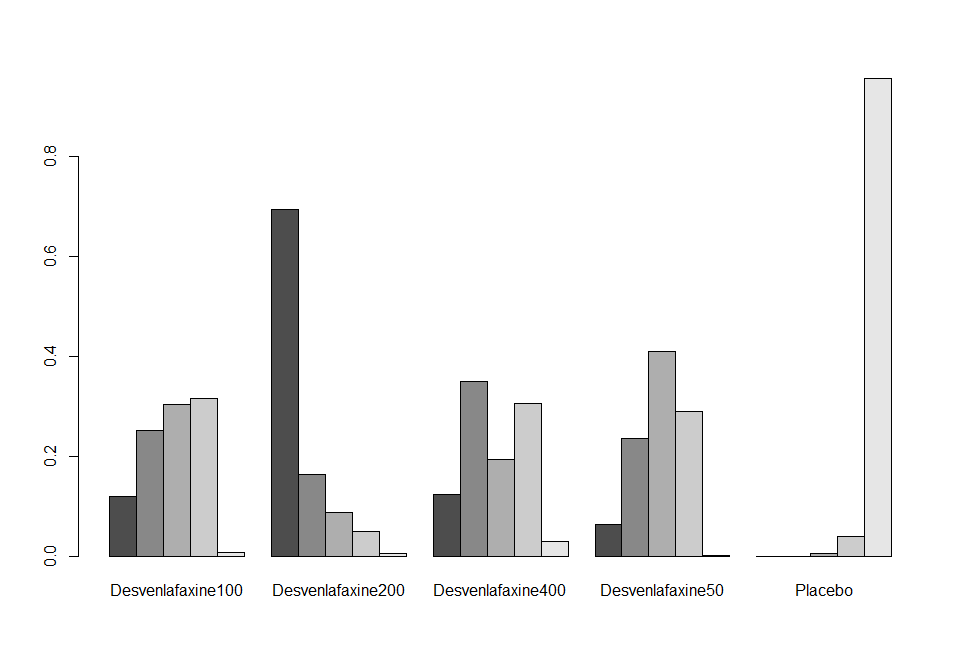


### League Table

|  | Desvenlafaxine100 | Desvenlafaxine200 | Desvenlafaxine400 | Desvenlafaxine50 | Placebo |
| --- | --- | --- | --- | --- | --- |
| Desvenlafaxine100 | Desvenlafaxine100 | -0.22 (-0.59, 0.16) | -0.02 (-0.37, 0.34) | 0.01 (-0.21, 0.22) | 0.38 (0.17, 0.59) |
| Desvenlafaxine200 | 0.22 (-0.16, 0.59) | Desvenlafaxine200 | 0.2 (-0.14, 0.53) | 0.22 (-0.1, 0.55) | 0.6 (0.29, 0.91) |
| Desvenlafaxine400 | 0.02 (-0.34, 0.37) | -0.2 (-0.53, 0.14) | Desvenlafaxine400 | 0.03 (-0.27, 0.33) | 0.4 (0.11, 0.69) |
| Desvenlafaxine50 | -0.01 (-0.22, 0.21) | -0.22 (-0.55, 0.1) | -0.03 (-0.33, 0.27) | Desvenlafaxine50 | 0.37 (0.28, 0.47) |
| Placebo | **-0.38 (-0.59, -0.17)** | **-0.6 (-0.91, -0.29)** | **-0.4 (-0.69, -0.11)** | **-0.37 (-0.47, -0.28)** | Placebo |

## Restricting to 8-week trials

### Sucra Ranking Diagram


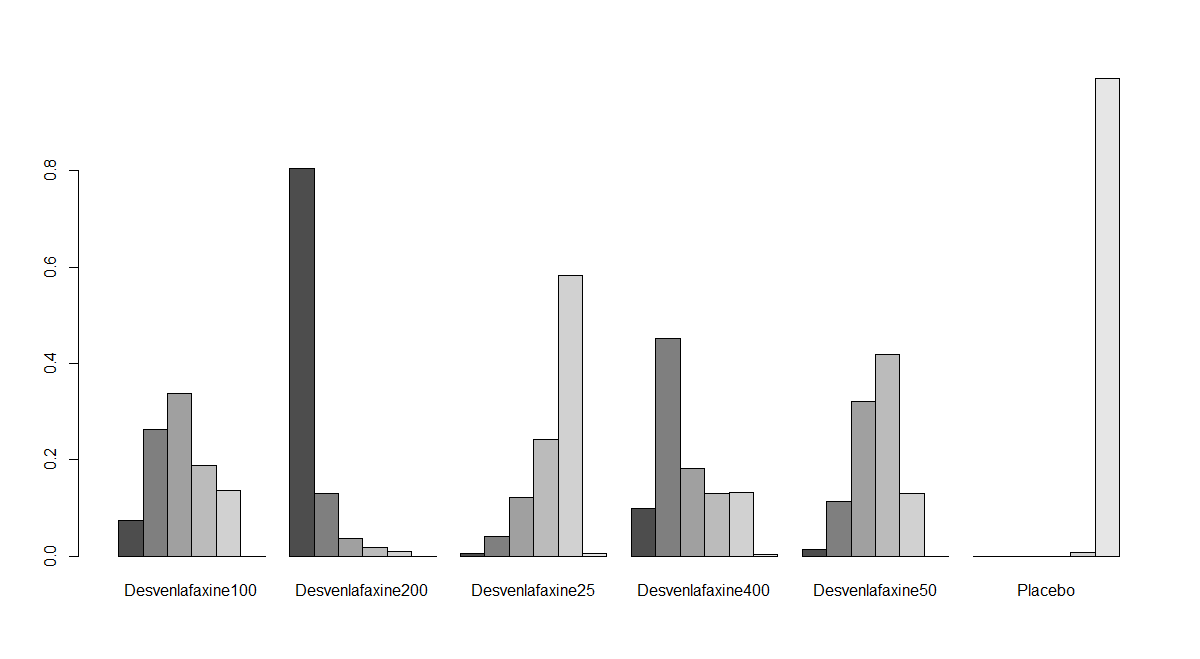


### League Table

|  | Desvenlafaxine100 | Desvenlafaxine200 | Desvenlafaxine25 | Desvenlafaxine400 | Desvenlafaxine50 | Placebo |
| --- | --- | --- | --- | --- | --- | --- |
| Desvenlafaxine100 | Desvenlafaxine100 | -0.24 (-0.62, 0.13) | 0.11 (-0.15, 0.38) | -0.05 (-0.4, 0.31) | 0.04 (-0.18, 0.26) | 0.35 (0.14, 0.57) |
| Desvenlafaxine200 | 0.24 (-0.13, 0.62) | Desvenlafaxine200 | 0.36 (0, 0.72) | 0.2 (-0.14, 0.53) | 0.29 (-0.05, 0.63) | 0.6 (0.29, 0.91) |
| Desvenlafaxine25 | -0.11 (-0.38, 0.15) | -0.36 (-0.72, 0) | Desvenlafaxine25 | -0.16 (-0.5, 0.18) | -0.07 (-0.25, 0.11) | 0.24 (0.06, 0.42) |
| Desvenlafaxine400 | 0.05 (-0.31, 0.4) | -0.2 (-0.53, 0.14) | 0.16 (-0.18, 0.5) | Desvenlafaxine400 | 0.09 (-0.23, 0.41) | 0.4 (0.11, 0.69) |
| Desvenlafaxine50 | -0.04 (-0.26, 0.18) | -0.29 (-0.63, 0.05) | 0.07 (-0.11, 0.25) | -0.09 (-0.41, 0.23) | Desvenlafaxine50 | 0.31 (0.17, 0.45) |
| Placebo | **-0.35 (-0.57, -0.14)** | **-0.6 (-0.91, -0.29)** | **-0.24 (-0.42, -0.06)** | **-0.4 (-0.69, -0.11)** | **-0.31 (-0.45, -0.17)** | Placebo |

# MADRS

## Excluding the 25 mg dose

### Sucra Ranking Diagram


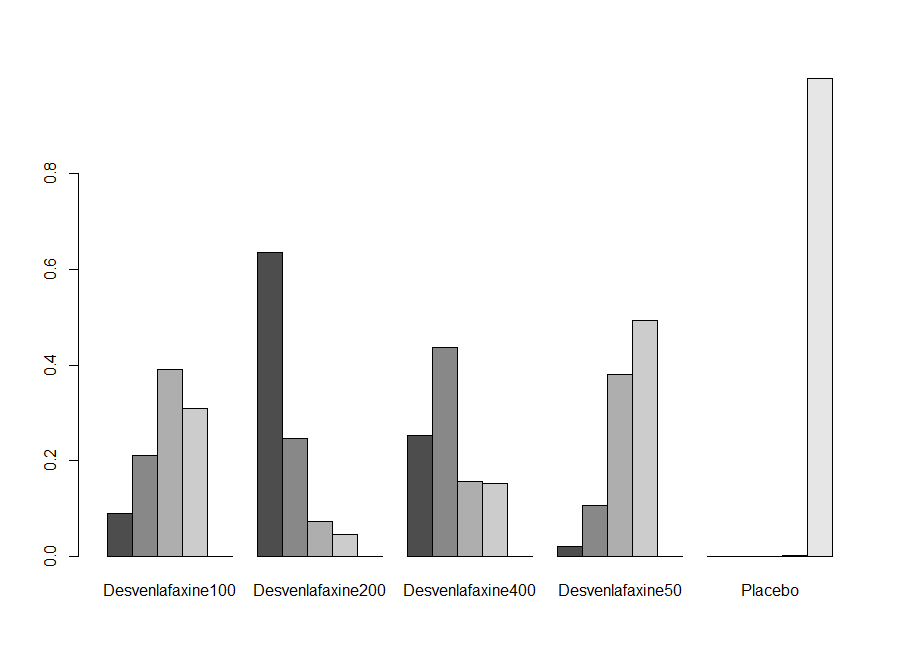


### League Table

|  | Desvenlafaxine100 | Desvenlafaxine200 | Desvenlafaxine400 | Desvenlafaxine50 | Placebo |
| --- | --- | --- | --- | --- | --- |
| Desvenlafaxine100 | Desvenlafaxine100 | -1.44 (-4.17, 1.29) | -0.75 (-3.46, 1.98) | 0.26 (-1.32, 1.84) | 2.85 (1.29, 4.43) |
| Desvenlafaxine200 | 1.44 (-1.29, 4.17) | Desvenlafaxine200 | 0.7 (-1.84, 3.24) | 1.71 (-0.73, 4.14) | 4.3 (2.06, 6.53) |
| Desvenlafaxine400 | 0.75 (-1.98, 3.46) | -0.7 (-3.24, 1.84) | Desvenlafaxine400 | 1.01 (-1.39, 3.41) | 3.61 (1.4, 5.81) |
| Desvenlafaxine50 | -0.26 (-1.84, 1.32) | -1.71 (-4.14, 0.73) | -1.01 (-3.41, 1.39) | Desvenlafaxine50 | 2.59 (1.63, 3.55) |
| Placebo | **-2.85 (-4.43, -1.29)** | **-4.3 (-6.53, -2.06)** | **-3.61 (-5.81, -1.4)** | **-2.59 (-3.55, -1.63)** | Placebo |

## Restricting to 8-week trials

### Sucra Ranking Diagram


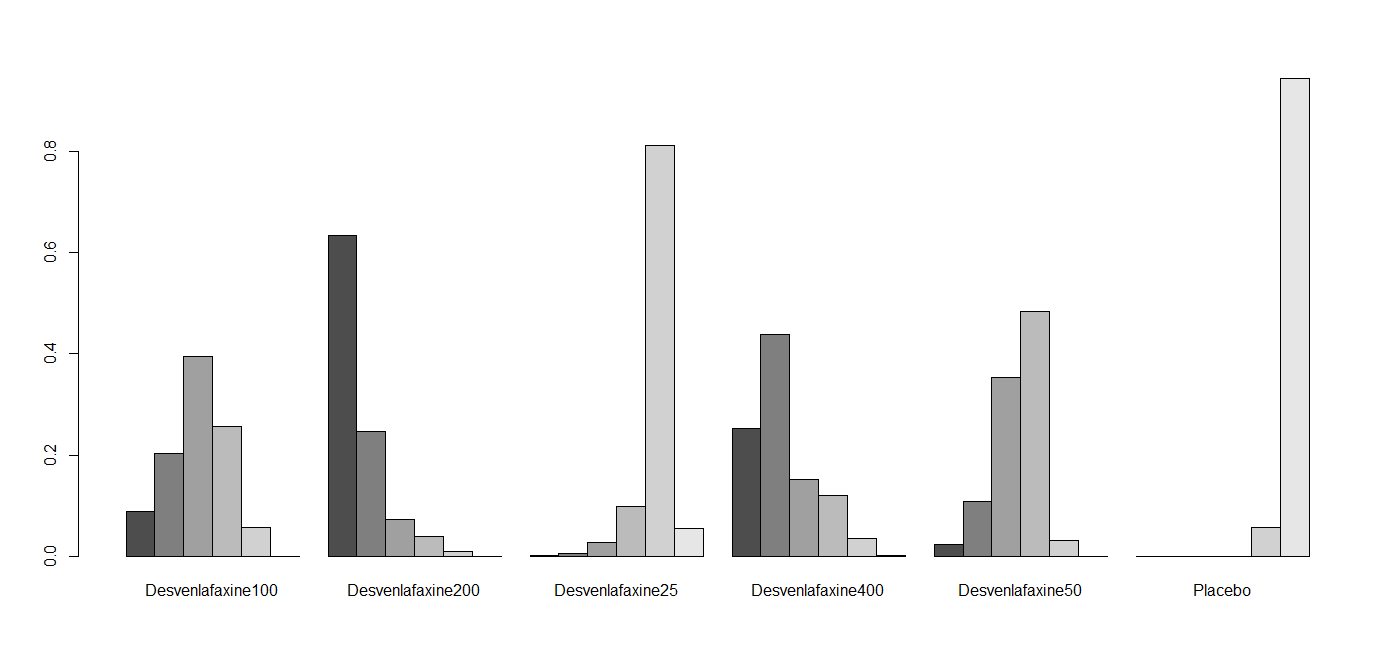


### League Table

|  | Desvenlafaxine100 | Desvenlafaxine200 | Desvenlafaxine25 | Desvenlafaxine400 | Desvenlafaxine50 | Placebo |
| --- | --- | --- | --- | --- | --- | --- |
| Desvenlafaxine100 | Desvenlafaxine100 | -1.45 (-4.18, 1.3) | 1.59 (-0.5, 3.67) | -0.75 (-3.48, 1.96) | 0.29 (-1.34, 1.91) | 2.84 (1.25, 4.45) |
| Desvenlafaxine200 | 1.45 (-1.3, 4.18) | Desvenlafaxine200 | 3.03 (0.32, 5.74) | 0.7 (-1.84, 3.22) | 1.73 (-0.76, 4.21) | 4.29 (2.06, 6.51) |
| Desvenlafaxine25 | -1.59 (-3.67, 0.5) | **-3.03 (-5.74, -0.32)** | Desvenlafaxine25 | -2.34 (-5.03, 0.35) | -1.3 (-2.85, 0.24) | 1.25 (-0.29, 2.8) |
| Desvenlafaxine400 | 0.75 (-1.96, 3.48) | -0.7 (-3.22, 1.84) | 2.34 (-0.35, 5.03) | Desvenlafaxine400 | 1.04 (-1.43, 3.51) | 3.59 (1.39, 5.8) |
| Desvenlafaxine50 | -0.29 (-1.91, 1.34) | -1.73 (-4.21, 0.76) | 1.3 (-0.24, 2.85) | -1.04 (-3.51, 1.43) | Desvenlafaxine50 | 2.56 (1.43, 3.67) |
| Placebo | **-2.84 (-4.45, -1.25)** | **-4.29 (-6.51, -2.06)** | -1.25 (-2.8, 0.29) | **-3.59 (-5.8, -1.39)** | **-2.56 (-3.67, -1.43)** | Placebo |

# TEAEs

## Excluding the 25 mg dose

### Sucra Ranking Diagram


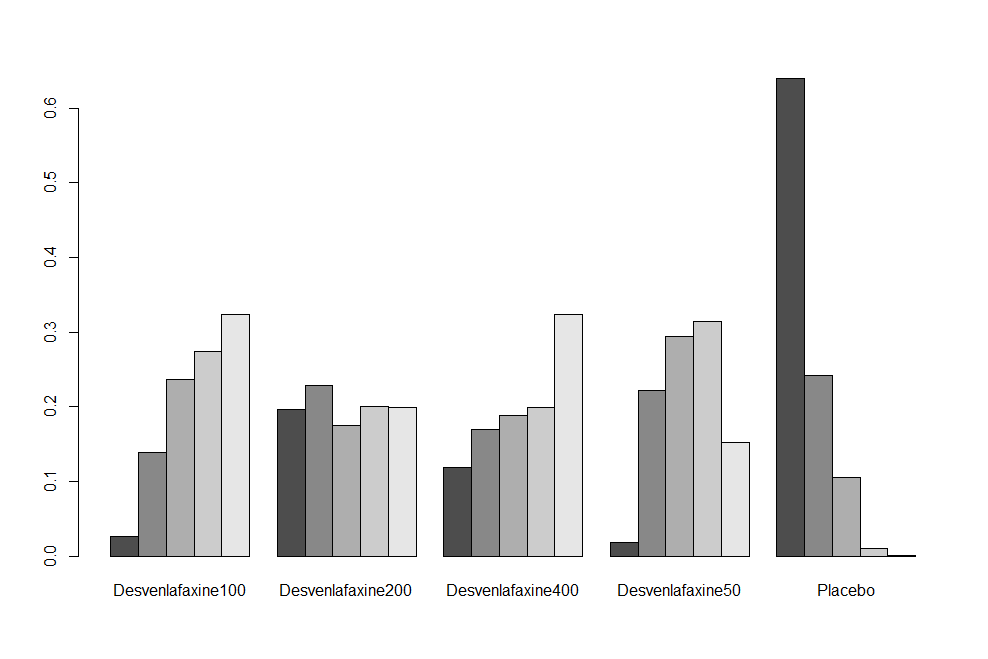


### League Table

|  | Desvenlafaxine100 | Desvenlafaxine200 | Desvenlafaxine400 | Desvenlafaxine50 | Placebo |
| --- | --- | --- | --- | --- | --- |
| Desvenlafaxine100 | Desvenlafaxine100 | 0.92 (0.44, 1.83) | 0.98 (0.47, 1.96) | 0.96 (0.68, 1.33) | 0.76 (0.54, 1.05) |
| Desvenlafaxine200 | 1.09 (0.55, 2.26) | Desvenlafaxine200 | 1.07 (0.58, 1.98) | 1.04 (0.54, 2.06) | 0.83 (0.45, 1.55) |
| Desvenlafaxine400 | 1.02 (0.51, 2.11) | 0.94 (0.51, 1.73) | Desvenlafaxine400 | 0.98 (0.5, 1.93) | 0.78 (0.42, 1.45) |
| Desvenlafaxine50 | 1.05 (0.75, 1.47) | 0.96 (0.48, 1.87) | 1.02 (0.52, 1.99) | Desvenlafaxine50 | 0.8 (0.61, 1.02) |
| Placebo | 1.31 (0.95, 1.87) | 1.2 (0.64, 2.24) | 1.28 (0.69, 2.39) | 1.25 (0.98, 1.63) | Placebo |

## Restricting to 8-week trials

### Sucra Ranking Diagram


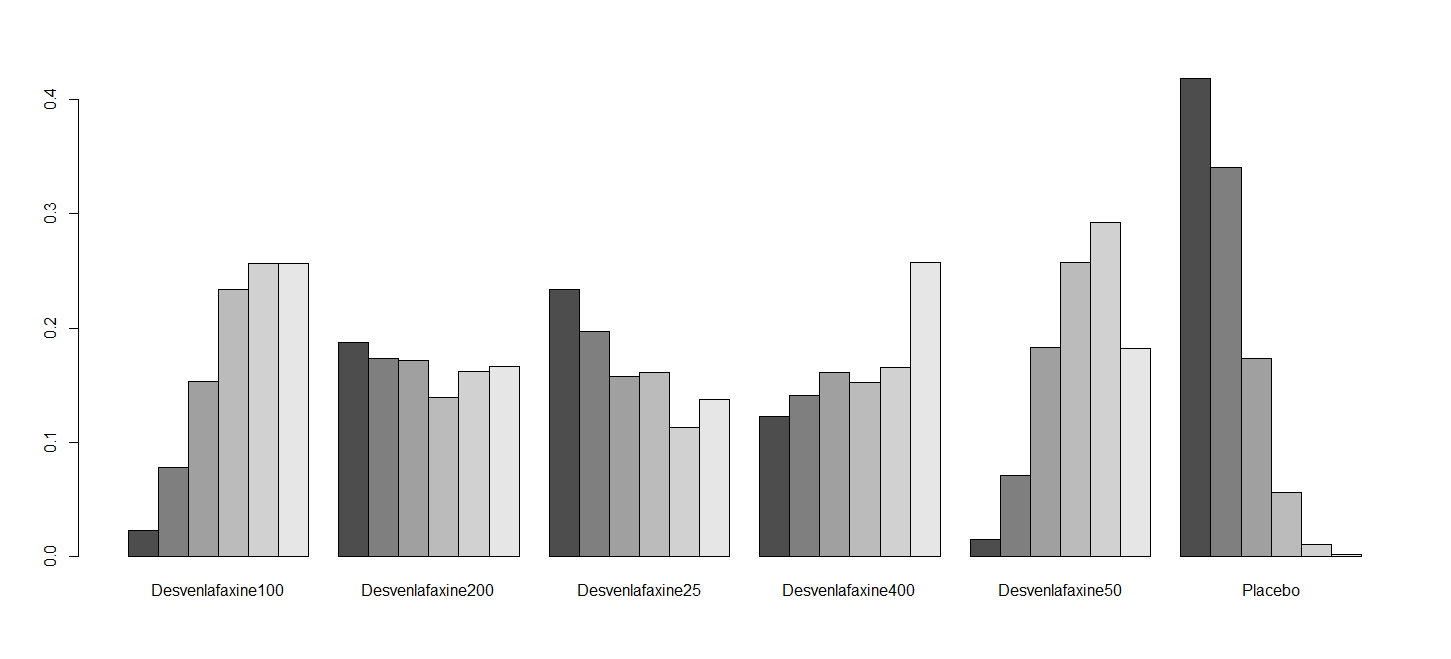


### League Table

|  | Desvenlafaxine100 | Desvenlafaxine200 | Desvenlafaxine25 | Desvenlafaxine400 | Desvenlafaxine50 | Placebo |
| --- | --- | --- | --- | --- | --- | --- |
| Desvenlafaxine100 | Desvenlafaxine100 | 0.88 (0.36, 2.11) | 0.85 (0.39, 1.82) | 0.94 (0.38, 2.26) | 0.98 (0.64, 1.51) | 0.73 (0.47, 1.12) |
| Desvenlafaxine200 | 1.13 (0.47, 2.81) | Desvenlafaxine200 | 0.96 (0.34, 2.76) | 1.07 (0.5, 2.31) | 1.11 (0.47, 2.7) | 0.83 (0.38, 1.81) |
| Desvenlafaxine25 | 1.18 (0.55, 2.59) | 1.04 (0.36, 2.94) | Desvenlafaxine25 | 1.12 (0.39, 3.13) | 1.16 (0.58, 2.35) | 0.87 (0.43, 1.74) |
| Desvenlafaxine400 | 1.06 (0.44, 2.62) | 0.94 (0.43, 2.01) | 0.9 (0.32, 2.57) | Desvenlafaxine400 | 1.04 (0.45, 2.52) | 0.78 (0.36, 1.69) |
| Desvenlafaxine50 | 1.02 (0.66, 1.57) | 0.9 (0.37, 2.12) | 0.86 (0.42, 1.72) | 0.96 (0.4, 2.25) | Desvenlafaxine50 | 0.75 (0.5, 1.09) |
| Placebo | 1.36 (0.89, 2.15) | 1.2 (0.55, 2.61) | 1.15 (0.58, 2.33) | 1.29 (0.59, 2.78) | 1.34 (0.92, 2.01) | Placebo |
